# Impacts of the Biocontrol Strain *Pseudomonas simiae* PICF7 on the Banana Holobiont: Alteration of Root Microbial Co-occurrence Networks and Effect on Host Defense Responses

**DOI:** 10.3389/fmicb.2022.809126

**Published:** 2022-02-15

**Authors:** Carmen Gómez-Lama Cabanás, Nuria M. Wentzien, Yasmín Zorrilla-Fontanesi, Antonio Valverde-Corredor, Antonio J. Fernández-González, Manuel Fernández-López, Jesús Mercado-Blanco

**Affiliations:** ^1^Departamento de Protección de Cultivos, Instituto de Agricultura Sostenible, Consejo Superior de Investigaciones Científicas, Córdoba, Spain; ^2^Departamento de Microbiología del Suelo y Sistemas Simbióticos, Estación Experimental del Zaidín, Consejo Superior de Investigaciones Científicas, Granada, Spain; ^3^Laboratory of Tropical Crop Improvement, Division of Crop Biotechnics, KU Leuven, Leuven, Belgium

**Keywords:** beneficial rhizobacteria, biocontrol agents, co-occurrence networks, defense-related genes, microbiome, *Musa acuminata*, plant–microbe interaction, *Pseudomonas simiae* PICF7

## Abstract

The impact of the versatile biocontrol and plant-growth-promoting rhizobacteria *Pseudomonas simiae* PICF7 on the banana holobiont under controlled conditions was investigated. We examine the fate of this biological control agent (BCA) upon introduction in the soil, the effect on the banana root microbiota, and the influence on specific host genetic defense responses. While the presence of strain PICF7 significantly altered neither the composition nor the structure of the root microbiota, a significant shift in microbial community interactions through co-occurrence network analysis was observed. Despite the fact that PICF7 did not constitute a keystone, the topology of this network was significantly modified—the BCA being identified as a constituent of one of the main network modules in bacterized plants. Gene expression analysis showed the early suppression of several systemic acquired resistance and induced systemic resistance (ISR) markers. This outcome occurred at the time in which the highest relative abundance of PICF7 was detected. The absence of major and permanent changes on the banana holobiont upon PICF7 introduction poses advantages regarding the use of this beneficial rhizobacteria under field conditions. Indeed a BCA able to control the target pathogen while altering as little as possible the natural host-associated microbiome should be a requisite when developing effective bio-inoculants.

## Introduction

Banana (*Musa* spp.), including the dessert banana and the cooking types or plantains, globally constitutes the most important and traded fruit, and its annual production reached 155 million metric tons in 2018 (FAOSTAT, 2020). The crop is grown in more than 120 countries across the tropics and subtropics, where around 400 million people are depending on it for food security and income. Cultivated bananas evolved from inter- and intra-specific hybridizations between two wild diploid ancestors, *Musa acuminata* (A genome) and *Musa balbisiana* (B genome) (Simmonds and Shepherd, 1955). These crosses produced edible bananas that are often triploids (genome configurations AAA, AAB, and ABB) and have parthenocarpic fruits (Ortiz and Swennen, 2014). Within the AAA group, the “Cavendish” subgroup includes the cultivars that dominate the international trade (e.g., Grand Nain) and, as such, have set the standards in terms of taste, yield, and post-harvest characteristics expected of an export banana.

Among the banana production constraints, Fusarium wilt, caused by the soil-borne fungal pathogen *Fusarium oxysporum* f. sp. *cubense* (*Foc*) (E.F. Smith) W. C. Snyder & H. N. Hansen, is considered one of the most destructive diseases for this crop worldwide. It has been classified into three physiological races, namely, Race 1 (R1), Race 2 (R2), and Race 4 (R4), although the latter was further divided into subtropical (STR4) and tropical (TR4) races (Buddenhagen, 2009). This classification is based on the host range of cultivars on which they cause disease, and at least 24 vegetative compatibility groups within the different races have been described (Mostert et al., 2017). *Foc* R1 causes disease in the cultivar Gros Michel, Silk, and Pome subgroup, among others; *Foc* R2 affects the same subgroups as R1 and also the Bluggoe subgroup; and R4 causes wilt on most cultivars, including the Cavendish subgroup (AAA genome) (Ploetz, 2015). Currently, TR4 constitutes the main threat to banana production due to its extreme virulence toward Cavendish cultivars, which constitute a monoculture in large-scale industrial plantations. Different management measures exist against *Foc*, but none of them, when applied individually, have provided complete protection to this disease (Ploetz, 2015; Dita et al., 2018; Kema et al., 2021). Therefore, efforts have focused on integrated management strategies that include, among other measures, the application of biological control agents (BCA) to reduce the impact of Fusarium wilt of banana (FWB) on susceptible cultivars or to increase the durability of those ones already tolerant (Köberl et al., 2017).

Mechanisms underlying biological control can have either a direct effect on the pathogen—for example, through antibiosis, parasitism, or competition for space and/or nutrients—or an indirect effect by stimulating host defense mechanisms such as local responses, systemic acquired resistance, or induced systemic resistance (ISR) (Bubici et al., 2019). Furthermore, indirect biocontrol activity can be achieved through the modulation of the microbiome living in close association with the plant and/or in the soil (Xue et al., 2015; Bubici et al., 2019; Berg et al., 2020). Consequently, the plant and its associated microbial communities constitute a meta-organism, the so-called holobiont, which usually is also an important reservoir of beneficial microorganisms, including BCA (Hassani et al., 2018). The plant-associated microbiome thus decisively contributes to the health, fitness, development, and productivity of the holobiont by, for instance, enhancing the tolerance to different (a)biotic stresses and/or promoting the growth of the host (Mendes et al., 2013; Berg et al., 2017; Fernández-González et al., 2021).

The biological control of FWB has proven to be a successful strategy, which was widely implemented in the management of this disease. The most common microbial genera with effective biocontrol activity against FWB are *Pseudomonas*, *Bacillus*, *Streptomyces*, *Trichoderma*, non-pathogenic *F. oxysporum*, and some arbuscular mycorrhizal fungi [Bubici et al. (2019) and references therein]. Even though most of these antagonists originated from the banana rhizosphere soil or banana roots, as native constituents of the root endophytome (Köberl et al., 2015, 2017; Liu et al., 2019; Zhou et al., 2019; Kaushal et al., 2020a,b), some of them were isolated from other plant species. This is the case of the strain *Pseudomonas simiae* PICF7 (formerly *Pseudomonas fluorescens* PICF7; Montes-Osuna et al., 2021), a highly versatile beneficial endophytic rhizobacteria isolated from olive roots, which display biocontrol and plant-growth-promoting abilities under different experimental conditions (Maldonado-González et al., 2013, 2014, 2015; Gómez-Lama Cabanás et al., 2014, 2018; Mercado-Blanco et al., 2016; Desrut et al., 2021). Recently, strain PICF7 proved to be an effective BCA against *Foc* STR4, displaying better FWB biocontrol than indigenous rhizobacteria isolated from banana roots (Gómez-Lama Cabanás et al., 2021). Although several BCAs mentioned above were capable of controlling FWB, our knowledge of the mechanisms involved is limited.

The microbiota present in a given plant holobiont forms a complex network whose components are continuously interacting among them (Wassermann et al., 2019) and with the host transcriptome (Fernández-González et al., 2021). Consequently, the application of a BCA will not only affect the host (e.g., triggering genetic defense responses) and/or the pathogen (e.g., antibiosis) but also likely have an impact on the composition, structure, and/or functionality of the host-associated microbiome. However, the potential effect of a given BCA over the natural microbiota has been less investigated (Massart et al., 2015; Roquigny et al., 2018; Berg et al., 2021; Zhimo et al., 2021). The impact of introducing an alien microorganism (either a pathogen or a BCA) on the holobiont microbiota can take place at different levels, such as the taxonomic profiles, structure, composition, functionality, and/or co-occurrence network topology. This has been recently highlighted in the case of the introduction of soil-borne pathogens (i.e., Fernández-González et al., 2020; Snelders et al., 2020). Nevertheless, there is still scant information on whether these changes take place upon BCA application and/or whether they can contribute to pathogen biocontrol. This is particularly true in the case of the interactions among components of the microbiota of the holobiont. Indeed co-occurrence network analyses could provide useful information about relevant members of the plant microbiota in order to improve plant health and counteract potential threats more effectively (Fernández-González et al., 2020; Zheng et al., 2021). This information can be helpful in order to develop bioformulations based on either a single microorganism or consortia, which must include the evaluation of potential impacts (risk assessment) on the target niche. Indeed although the introduction of BCA always causes effects on the holobiont, it would be desirable to apply them while altering as little as possible the natural plant/soil microbiome, thus avoiding major imbalances in the system (Köhl et al., 2019). In this context, changes in the plant-associated microbiome due to the introduction of a BCA and the identification of genes associated to *Foc* resistance/tolerance would also help in designing better strategies for FWB management (Zorrilla-Fontanesi et al., 2020). Several studies have reported that BCAs are able to trigger banana plant defense responses against *Foc* which are associated with the control of FWB (Fishal et al., 2010; Thangavelu et al., 2016; Savani et al., 2020). However, the mechanisms involved in the control of FWB by *P. simiae* PICF7 still remains to be elucidated.

The aim of this research is to assess the impact of the biocontrol strain *P. simiae* PICF7 on the banana holobiont. We therefore pursue a double objective: (i) to unravel the structure, composition, and co-occurrence network interactions of the microbial communities associated to banana [cv. Grand Nain (GN), Cavendish subgroup] roots in the absence/presence of the BCA and (ii) to investigate the ability of this beneficial rhizobacteria to trigger specific local and/or systemic defense genetic responses in the host.

## Materials and Methods

### Plant Material and *Pseudomonas simiae* PICF7 Inoculation

*In vitro*-propagated banana plants, GN Cavendish cultivar, kindly provided by CULTESA (Cultivos y Tecnología Agraria de Tenerife S.A, Tacoronte, Sta. Cruz de Tenerife, Spain), were used in this study. On the one hand and in order to examine the pre-existing root microbiota, roots from 10 Grand Nain *in vitro* (GNI) plants were sampled before being transplanted into pots. Thus, the roots were directly frozen at -80°C for later DNA extractions. On the other hand, an additional set of 40 GN banana plants were transplanted into polypropylene pots (11 cm × 11 cm × 12 cm, one plant per pot), each containing an *ad hoc*-prepared non-sterile substrate made of peat/sand/vermiculite (1:1:1 v/v/v). The pots were randomly distributed (four plants per time-point and per treatment) in a growth chamber under artificial lighting (14-h photoperiod of fluorescent light at 360 μE/m^2^) and subtropical conditions (26–20°C, humidity 65%). Prior to the bacterial treatment, the plants were acclimated for approximately 7 weeks (until the plants developed four or five true leaves) in the same growth chamber where the experiment was carried out. The *P. simiae* PICF7 inoculum was prepared as described by Gómez-Lama Cabanás et al. (2021). Twenty Grand Nain *in vitro* banana plants were inoculated (GNF7) by adding 150 ml of a suspension of bacterial cells (1 × 10^8^ cfu/ml in sterile MgSO_4_⋅7H_2_O 10 mM) per pot. Grand Nain control (GNC) plants (20) were just drenched with 150 ml of sterile MgSO_4_⋅7H_2_O 10 mM. After that, the roots were carefully uprooted from the original substrate and cleaned manually in order to remove most of the substrate particles adhered to the roots. Therefore, the root tissues and the substrate firmly adhered to the rhizoplane of each banana plant were sampled at 1, 2, 7, 15, and 56 (four plants per time-point/treatment) days after PICF7 inoculation, snap-frozen in liquid nitrogen, and stored at -80°C until the DNA and RNA extractions. The same plants were used for analyzing the banana root microbiome and the gene expression.

### DNA Extraction and Illumina Sequencing

Each individual root sample was disrupted by grinding in liquid nitrogen. The DNA from 100 mg of ground root tissues was extracted using the Maxwell RSC (Rapid Sample Concentrator) with the “PureFood GMO and Authentication” Kit (Promega Corporation, Madison, WI, United States) according to the instructions of the manufacturer. DNA yield and quality were checked both by electrophoresis in 0.8% (w/v) agarose gels stained with GelRed and visualized under UV light and by using a Qubit 3.0 fluorometer (Life Technologies, Grand Island, NY, United States). The DNA from root tissues was sequenced using the Illumina MiSeq platform at the genomics service of the Institute of Parasitology and Biomedicine “López Neyra” (CSIC), Granada, Spain. In the first run, prokaryotic libraries were constructed by amplifying the hyper-variable V3 to V4 regions of the *16S rRNA* gene, and in the second run, eukaryotic libraries were constructed by amplifying the ITS2. The primer pairs used and additional information concerning the sequencing process are detailed by Gómez-Lama Cabanás et al. (2021).

### Illumina Data Processing

Raw reads were processed using DADA2 (Callahan et al., 2016). The pipeline tutorials for *16S rRNA* amplicon processing^1^ and for ITS2 amplicon processing^2^ were followed. Some parameters were modified according to our data as detailed below. Only one sample (i.e., GNI06 belonging to the group of plants sampled before inoculation with strain PICF7) had to be eliminated from the analysis. First of all, the primers of both datasets were removed in a different way, according to the recommendations of the above-mentioned tutorials (Callahan et al., 2016). The bacterial primers were removed simultaneously in the trimming step using the *trimLeft* argument of the *filterAndTrim* command. In this step, default parameters were used, with the exception of *truncLen* (set to 280 nt for forward reads and 240 nt for reverse reads, according to their quality profiles) and *maxEE* (set to two and five maximum expected errors for forward and reverse reads, respectively). Then, the R1 and R2 reads were merged, and default parameters were used. Before chimera detection, merged reads smaller than 400 nt and larger than 480 nt were discarded. Similar steps were followed for fungal data processing, although no *truncLen* was used as recommended in the tutorial. After filtering with *maxEE* set to two and five and merging with default parameters, reads smaller than 100 nt were removed. In both bacterial and fungal data, chimeras were detected and discarded with the *removeBimeraDenovo* command. The classification of bacterial and fungal amplicon sequence variants (ASV) was achieved using the *assignTaxonomy* command against the Ribosomal Database Project II, training set v.16 (Cole et al., 2014), and the UNITE v.7.2 dynamic database,^3^ respectively. All ASV classified as mitochondria, chloroplast, and unknown sequences were removed, and only ASV classified as fungi at the kingdom level were considered for further analysis. Finally, ASV accounting for less than 0.0028 and 0.005% of the total sequences were removed for bacteria and fungi, respectively; such percentages have been calculated according to the MOCK community used [ZymoBIOMICS Microbial Community Standard II (Log Distribution), ZYMO RESEARCH, CA, United States] and to Bokulich et al. (2013) recommendations. However, a special procedure was implemented to avoid discarding a great part of ASV of the GNI banana plants sampled before inoculation with strain PICF7. These *in vitro* samples had a much lower number of sequences than the banana roots sampled after potting (GNC and GNF7 samples). Thus, some ASV that would have been abundant enough in the GNI samples if processed independently did not reach the cutoff when analyzed together with the GNC and GNF7 root samples. In order to solve this bias, ASV not reaching the number of sequences according to the cutoff percentage were removed unless they were present in any GNI samples.

### Analysis of Alpha and Beta Diversities

Alpha diversity indices [observed richness; Shannon and inverse of Simpson diversity (InvSimpson)] were compared in rarefied samples with two-way analysis of variance (ANOVA) using the R package *car* (Fox and Weisberg, 2018) and with the Kruskal–Wallis test due to the lack of normality/homoscedasticity in some of these indices. *P*-values were false discovery rate (FDR)-corrected by the Benjamini–Hochberg method using the R package *agricolae* (de Mendiburu, 2017). As *post hoc* tests, Tukey honestly significant difference (HSD) and Wilcoxon signed-rank test were used to obtain ANOVA and Kruskal–Wallis results, respectively.

Concerning β-diversity, filtered ASV sequence counts were analyzed as previously described by Gómez-Lama Cabanás et al. (2021). Moreover, multiple-group comparisons were performed in order to elucidate biologically relevant and statistically significant variations between GNC and GNF7 plants at each sampling time-point in prokaryotic and fungal phyla and genera. This was performed using proportions in non-normalized counts with the STAMP v.2.1.3 software, selecting ANOVA Games–Howell’s *post hoc* test parameters. Besides this, R package *edgeR* was employed to reveal which ASV had a statistically significant different abundance between the conditions studied (two-group comparisons) using likelihood ratio tests. *P*-values were Benjamini–Hochberg FDR-corrected and considered significant when <0.05.

### Identification and Time-Course Changes in the Relative Abundance of *Pseudomonas simiae* PICF7 Amplicon Sequence Variants

For the purpose of analyzing the bio-inoculant behavior along time, it was necessary to identify which ASV corresponded to the *P. simiae* PICF7 inoculum. This identification was possible by using the web tool BLASTn.^4^ First, ASV classified as *Pseudomonas* in our analysis were identified. The ASV numbers with their corresponding sequences were stored hereunder as a FASTA file using the R package *seqinr* (Charif and Lobry, 2007). Afterward, this file was submitted in order to perform BLASTn against taxon sequences belonging to *P. simiae* (taxonomy ID: 321846), making use of the nucleotide collection (nr/nt) databases (Supplementary Table 4). The obtained results were analyzed for each ASV, identifying those ones with an exact match (100% identity) with the *16S rRNA* gene sequence of strain PICF7. Finally, these data were used to monitor the relative abundance of the identified PICF7 ASV found at all time-points after inoculation with the BCA.

### Banana Root Microbiota Network Construction

Microbial (bacterial and fungal) networks were separately constructed for control plants and plants inoculated with strain PICF7. Based on the results obtained after the PICF7 time-course change analysis, only samples from the first four time-points [1, 2, 7, and 15 days after inoculation (DAI)] and their corresponding replicates (*n* = 16 for each of both the control and the inoculated plants) were considered for every network. To build these networks, MENAP website was used^5^ following the instructions of the developer (Shannon et al., 2003; Zhou et al., 2010, 2011; Deng et al., 2012) and the detailed procedures and modifications described in Gómez-Lama Cabanás et al. (2021). However, in this study, the prevalence of the selected ASV was 50% as default.

### RNA Extraction, Purification, and cDNA Synthesis

Total RNA was extracted from banana roots and leaves sampled at 1, 2, 7, 15, and 56 DAI. Four plants per time-point and treatment (i.e., GNF7 vs. control GNC) were used. RNA extractions were carried out using the RNeasy Plus Mini Kit (Qiagen, Hilden, Germany), according to the instructions of the manufacturer but with the addition of polyvinylpyrrolidone 40,000 (VWR International, Radnor, PA, United States) to the lysis buffer at a final concentration of 5 mg/ml. A homogenization step was performed with a motorized pestle mixer (VWR^®^Pellet Mixer, VWR, Intl., Radnor, PA, United States). The samples were treated with TurboTM DNase I (Life Technology, Invitrogen, Carlsbad, CA, United States) for 45 min at 37°C followed by a phenol-chloroform/ethanol purification step to eliminate gDNA traces. Real-time qPCR was performed using DNase-treated RNA as template and primers for the *Elongation factor 1*α (*EF1*) genomic sequence in order to check the absence of gDNA. In the case that amplification was still detected after 40 cycles, an extra purification step was carried out until all gDNA traces were removed. The RNA yield and quality (A260/230, A260/280) were determined using Nanodrop ND-1000TM spectrophotometer (Thermo Fisher Scientific, Wilmington, DE, United States) and Qubit 3.0 fluorometer (Life Technologies, Grand Island, NY, United States). Synthesis of cDNA was performed from 500 ng of total RNA by using an oligo(dT)18 primer and the RevertAid H Minus First Strand cDNA Synthesis kit (Thermo Fisher Scientific, Inc., Waltham, MA, United States) following the instructions of the manufacturer with some modifications. First, incubation was performed at 70°C instead of 65°C, and the reaction was stopped after extending for five more minutes.

### Design and Optimization of RT-qPCR Primers

In this study, a paralogous inclusive expression approach (Cenci et al., 2019) was followed since banana experienced recent whole-genome duplications, and a large proportion of genes belongs to multigene families comprised by several paralogs, i.e., genes with highly similar DNA sequences (D’Hont et al., 2012; Cenci et al., 2014). In total, 13 genes were selected to examine their expression pattern in banana plants during a 56-day interval upon *P. simiae* PICF7 inoculation. From them, 11 genes are related to defense responses, and two genes are involved in reactive oxygen species (ROS) detoxification (Supplementary Table 5). After retrieving the *Arabidopsis thaliana* sequences of these genes from TAIR database,^6^ a protein blast analysis was conducted in Phytozome (Goodstein et al., 2012) in order to identify putative *M. acuminata* paralogs. Subsequently, those *M. acuminata* sequences showing the best blast result (lower e-value) were subjected to the Integrative Orthology Viewer analysis tool in PLAZA (Van Bel et al., 2018) to select, based on prediction methods, the genes that are most probably true paralogs of the previously identified Arabidopsis *thaliana* orthologs(s) (Supplementary Table 5). Paralogous sequences were downloaded from the *M. acuminata* v2 reference genome (Martin et al., 2016), accessible through the Banana Genome Hub (Droc et al., 2013). The primers were designed to detect global (combined) expression by targeting the most conserved region in the ORF and, thus, simultaneously amplifying all *M. acuminata* paralogs in each gene family. Primer 3 software version 0.4.0^7^ and Oligo Analyzer tool (Integrated DNA Technologies, Inc., Coralville, IA, United States) were used to find the most suitable primers. The primer combinations were custom-ordered from a commercial supplier (Integrated DNA Technology, Coralville, IA, United States) and tested at two concentrations (100 and 150 nM) by gradient PCR with gDNA. The amplicon sizes were checked by 2% agarose gel electrophoresis and ethidium bromide staining.

### Time-Course Gene Expression Analyses

Real-time qPCR runs were performed in a thermal cycler Bio-Rad CFX 384 real-time PCR detection system. The relative expression for each studied gene was repeated at least once for each biological replicate in independent real-time qPCR assays (plates), and three technical replicates per sample and per plate were always included. The RT-qPCR design, calculations, and statistics used followed the MIQE guidelines (Bustin et al., 2009). Each real-time qPCR reaction was carried out containing 2 μl of a 20 × diluted template cDNA, 0.3 μM of each primer, 5 μl of SYBR^®^ Green Supermix (BioRad, Hercules, CA, United States), and dH_2_O up to a total volume of 10 μl. Moreover, λ-DNA (5 ng ml^–1^) was added to the cDNA samples as carrier DNA to minimize absorption and Poisson effects. The following thermal cycling profile was used for all PCR reactions: 95°C for 10 s, 39 cycles of 95°C for 15 s, 60°C for 30 s, and 72°C for 15 s. The melting curves of real-time qPCR products were assessed from 65 to 95°C to verify the specificity of the reactions under the following conditions: initial denaturation for 10 s at 95°C, cooling to 65°C for 5 s, and melting from 65 to 95°C with 0.5°C transition rate every 10 s. Standard curves were generated for each selected gene using five serial fourfold dilutions of sample-pooled cDNA in order to calculate the gene-specific amplification efficiencies (*E*), correlation coefficients (*R*^2^), and corresponding linear equations (Supplementary Table 5). Data resulting from real-time qPCR were normalized to the *M. acuminata Ribosomal protein L2* (*L2*) and *Elongation factor 1*α (*EF1*α) genes (Zorrilla-Fontanesi et al., 2016). To determine the gene expression levels, a baseline was set manually, and quantification cycles (Cq) values were converted into calibrated normalized relative quantities using the RT-qPCR analysis software qBASE + (Biogazelle; Zwijnarde, Belgium^8^) and the method described by Hellemans et al. (2007). To examine the normality of gene expression data, the Shapiro–Wilk test was applied using STATISTICA 7.0 software (StatSoft, Inc., Tulsa, OK, United States). Data were multiplied by a common factor (10^3^) and base10-log-transformed either to reduce skewness and fit a normal distribution or to improve normality. When normality could not be improved, non-transformed data were used for statistical analyses and graphical representation. Subsequently, Fisher’s least significant difference mean comparison (*p* < 0.05) was applied using STATISTICA 7.0 in order to compare inoculated (GNF7) *vs.* control (GNC) conditions across all the analyzed time-points.

## Results

### General Characteristics of Sequencing Datasets

A total of 3,475,852 (bacterial) and 3,252,399 (fungal) raw reads were obtained by MySeq high-throughput sequencing. Only 2,028,422 (bacterial) and 2,160,942 (fungal) good-quality reads were finally retained after the clustering, representing at least 17,153 and a maximum of 69,061 sequences per sample from the prokaryotic dataset as well as 26,063 and 79,865 sequences from the fungal dataset, respectively. To avoid an overestimation of the diversity, ASV with less than 0.0028 and 0.005% of the high-quality bacterial and fungal reads, respectively, were discarded. A total of 1,839 bacterial and 415 fungal ASV were considered after all checking steps were carried out.

### Presence of Indigenous Microbiota in the Roots of *in vitro*-Propagated Banana (cv. Grand Nain) Plants

In order to determine whether the GNI-propagated plants used in this study harbor a natural microbial community in their roots, this material was examined before being transferred into the potting substrate. It is worth mentioning that, although GNI plants contained an associated microbiome, a high variability was found among samples in addition to very low diversity and species richness. Thus, taxonomical profiles for bacterial and fungal datasets are presented at the ASV level (Supplementary Table 1). Only ASV that were present in at least four or more samples were considered for analysis. Regarding the bacterial profile, the five most abundant ASV accounted for 12.64% of the mean relative abundance, and they were classified within the genera *Sphingomonas*, *Halomonas*, and *Mycobacterium* as well as two unclassified bacteria. With respect to fungal taxonomical composition, the five most abundant ASV accounted for 66.14% of the mean relative abundance, corresponding to the genera *Pseudogymnoascus*, *Geomyces*, *Mycosphaerella*, *Cladosporium*, and one unclassified fungi.

### Inoculation With *Pseudomonas simiae* PICF7 Modified Neither the Diversity nor the Structure of the Banana Root Microbiota

**FIGURE 1:**
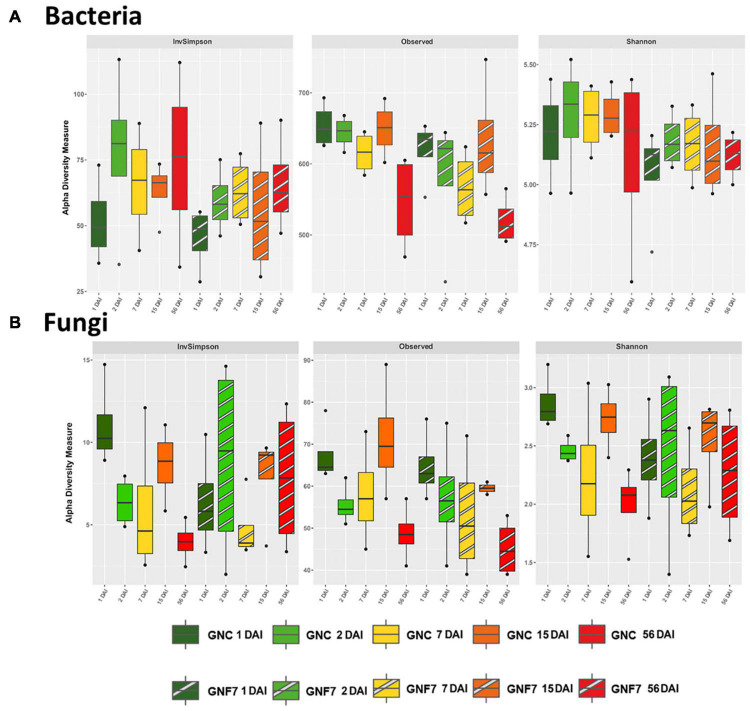
Box plot of α-diversity indices: InvSimpson, observed amplicon sequence variants, and Shannon for the bacterial **(A)** and fungal **(B)** communities of control (GNC) and *Pseudomonas simiae* PICF7-inoculated (GNF7) plants at different time-points (days after inoculation, DAI).

With the aim of elucidating the influence of PICF7 on the banana (cv. GN) root microbial community (rhizosphere and endosphere), non-inoculated (control, GNC) and PICF7-inoculated (GNF7) samples were firstly compared at each time-point collected after BCA inoculation. Concerning α-diversity, no significant differences were found for both bacterial and fungal datasets (Figure 1). Although species richness was significantly different according to ANOVA test (Supplementary Table 2, *p*-value 0.014 for bacteria and 0.018 for fungi), none of the pairwise comparisons between the GNC and GNF7 samples retrieved significant results. With regard to β-diversity and despite the fact that the PERMANOVA test showed statistically significant differences among groups (Supplementary Table 3) for both domains, none of the pairwise comparisons were significant. In order to confirm that this was not a consequence of variations in data dispersion, BETADISPER test was carried out, confirming that the variances were not statistically different. Despite the lack of significant pairwise comparisons, the bacterial PCoA on Bray–Curtis distances showed a trend, though non-significant, grouping all samples collected at 56 DAI without differences between GNC and GNF7 treatments (Supplementary Figure 1).

To avoid a bias due to variations within GNC samples or within GNF7 samples during the time-course of the experiment in the above-mentioned comparison, α-diversity and β-diversity analyses were also performed separately for GNC and GNF7 samples along time. Regarding α-diversity, significant differences were found neither for GNC nor GNF7 samples for bacterial dataset. Nonetheless, there was a trend for lower richness in samples collected at 56 DAI in both treatments (Figure 1). Interestingly, this trend was confirmed as statistically significant for fungal GNC samples. Specifically, fungal GNC diversity was significantly lower at 56 DAI in comparison to 1 DAI according to Shannon and InvSimpson indices. Moreover, species richness was also lower at 56 DAI in comparison to 15 DAI. It must be mentioned that this outcome was not observed for the mycobiota present in the GNF7 samples. In addition, no differences were observed in β-diversity pairwise comparisons for both domains and treatments, although PERMANOVA yielded statistically significant results, when comparing all the time together, and BETADISPER did not show significant variations in data dispersion (Supplementary Table 3).

The potential effect on the indigenous root microbial community present in *in vitro*-propagated plants (see previous section) after their transfer into the potting substrate was also examined by comparing the GNI treatment with the GNC and GNF7 samples. It must be emphasized that α-diversity analysis could not be carried out for the bacterial dataset due to sampling depth biases. Indeed the GNI samples showed a considerably lower number of sequences and species richness (observed ASV) in contrast to the GNC and GNF7 samples. Due to this fact, the process of rarefying samples to the lowest sequence number made it impossible to reach the asymptote for the GNC and GNF7 samples (Supplementary Figure 2), thus producing a great loss of information. This was not the case for the fungal dataset. Indeed the results showed that fungal richness was significantly lower for the GNI samples in comparison with both GNC and GNF7 treatments, in accordance with ANOVA and Tukey HSD post hoc tests (Supplementary Figure 3 and Supplementary Table 2). In relation to β-diversity, clear differences were found between the GNI and GNC/GNF7 samples for both domains (Supplementary Figure 4). The β-diversity pairwise comparisons for both domains resulted in GNI samples being significantly different from GNC and GNF7 samples, as expected from ordination plots.

### Taxonomical Composition of the Banana Root Microbiota in the Absence/Presence of *Pseudomonas simiae* PICF7

**FIGURE 2:**
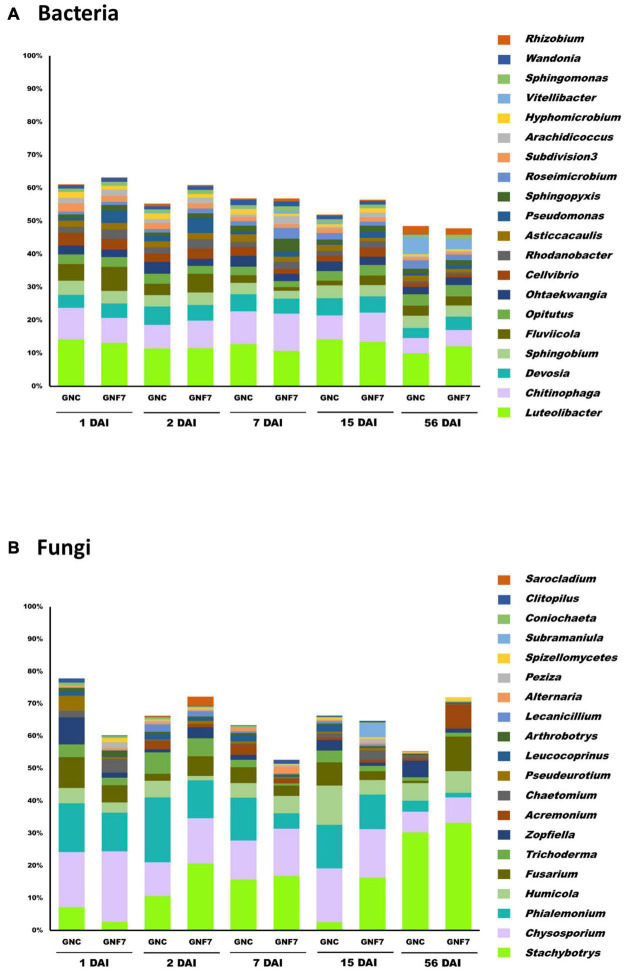
Bacterial **(A)** and fungal **(B)** taxonomic profiles at the genus level of banana (cv. Grand Nain) roots non-inoculated (GNC) and inoculated (GNF7) with strain PICF7 at 1, 2, 7, 15, and 56 days after inoculation (DAI). Only the main genera (with a relative abundance ≥1%) are shown (*n* = 4).

Concerning the GNC and GNF7 samples, bacterial taxonomical profile at the phylum level was dominated by Proteobacteria, Bacteroidetes, Verrucomicrobia, Actinobacteria, Acidobacteria, Planctomycetes, and Armatimonadetes. These phyla accounted for at least 85.8% of all sequences. At the genus level, *Luteolibacter*, *Chitinophaga*, *Devosia*, *Sphingobium*, *Fluviicola*, and *Opitutus* showed the highest relative abundance (Figure 2A). Significant differences were not found either at the phylum or the genera levels between GNC and GNF7 at any time-point after inoculation with the BCA.

With regard to the fungal dataset, Ascomycota was the most abundant phylum, representing no less than 92.56% of the relative abundance. Thus, the most abundant genera in the GNC and GNF7 samples were *Stachybotrys*, *Chrysosporium*, *Phialemonium*, *Humicola*, and Fusarium (Figure 2B). Although a few genera (e.g., *Stachybotrys*, *Chrysosporium*, or *Phialemonium*) seemed to differ in relative abundance between groups (Figures 2A,B), no significant differences were found when assessing two-group comparisons between GNC and GNF7 at each time-point.

Moreover, differences were checked at the ASV level. In accordance with the α-diversity, β-diversity, and taxonomical profiling results, the two-group comparisons at each time-point sampled only showed differences for minor ASV. Regarding the bacterial dataset, these ASV did not account for more than 1% of the sequences in any of the time-points sampled, except for ASV024 and ASV054. Interestingly enough, ASV024 was classified as *Pseudomonas* at the genus level and was present in the GNF7 samples but absent in the GNC samples at all the studied time-points. Concerning ASV054, it was classified as *Opitutus*, and the only difference was found at 7 DAI comparison. With respect to the fungal dataset, only ASV08, classified within the genus *Stachybotrys*, showed significant differences, with this ASV being more abundant in GNF7 at 56 DAI and totally absent in GNC at this time-point.

Finally, despite the drastic change in the microbial communities of GNI plants due to substrate transfer, some ASV such as ASV056, classified as *Mycobacterium*, were still present in the GNC and GNF7 samples at all the time-points sampled. A similar finding was observed for two fungal genera. Thus, ASV060 (*Cladosporium*) and ASV09 (*Phialemonium*), already present in the GNI samples, were also identified in the GNC and GNF7 samples during the time-course of the experiment.

### Identification of *Pseudomonas simiae* PICF7 and Time-Course Changes in Its Relative Abundance

**FIGURE 3:**
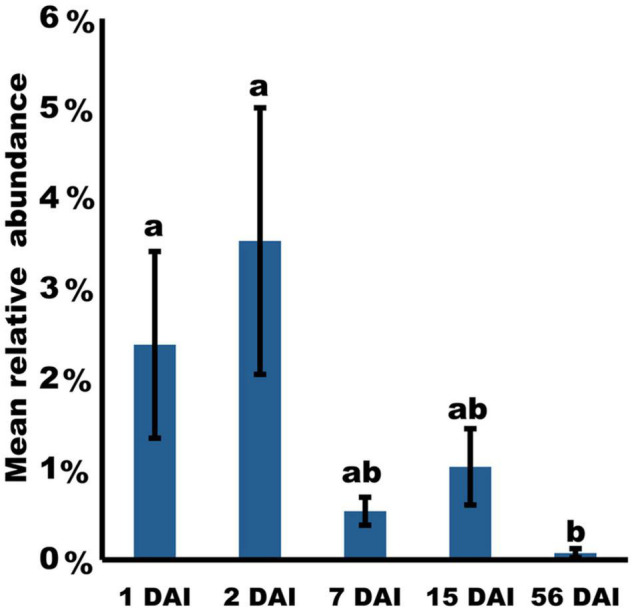
Time-course changes in relative abundance of *Pseudomonas simiae* PICF7 (ASV024) in banana roots during an interval of 56 days based on *16S rRNA* gene amplicon sequence. *P*-values were calculated using likelihood ratio test. Different letters indicate significant differences (*p* < 0.05). Error bars indicate standard deviations (*n* = 4).

Among all ASV found, 37 ASV were classified as belonging to the *Pseudomonas* genus. In order to identify the presence of PICF7 in the samples, sequences of these 37 ASV were additionally checked using the BLASTn online tool. Interestingly, ASV024 was the only one showing a perfect match with the *P. simiae* PICF7 (GenBank: CP005975.1) *16S rRNA* gene sequence (Supplementary Table 4). Besides this, ASV024 was significantly more abundant in the GNF7 samples during the first days after PICF7 bacterization, with relative abundances at 1 and 2 DAI (2.38 and 3.53% of the average number of bacterial sequences, respectively) being significantly higher in comparison with the samples collected at 56 DAI (*p*-value = 0.013 and *p*-value = 0.011, respectively) (Figure 3). Furthermore, ASV024 was completely absent in the GNC samples as expected for the PICF7-free (control) samples.

### Strain PICF7 Altered the Banana Root Microbial Co-occurrence Network

**FIGURE 4:**
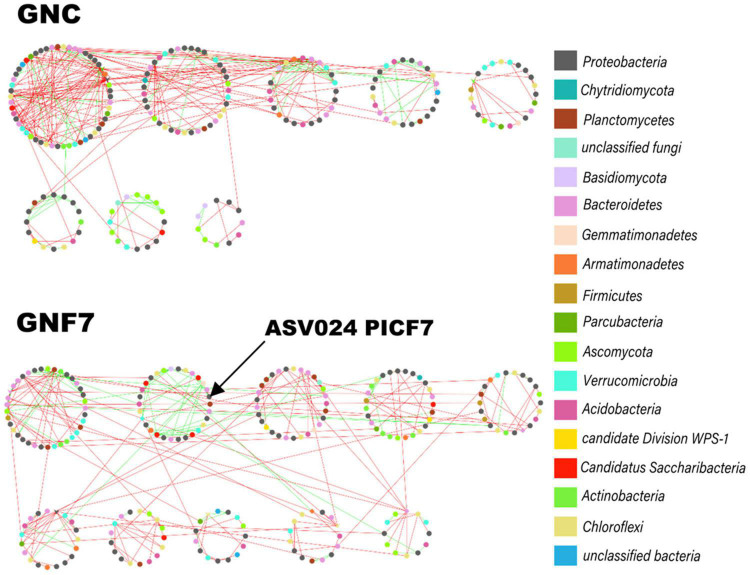
Co-occurrence networks of microbial communities in banana (cv. Grand Nain) root control (GNC) and *Pseudomonas simiae* PICF7-inoculated (GNF7) plants. This panel shows the modular layout of the networks, with the nodes colored according to their phylum. The green and red lines (links) indicate positive and negative interactions, respectively. The arrow indicates module position in which the PICF7 amplicon sequence variant was found.

**FIGURE 5:**
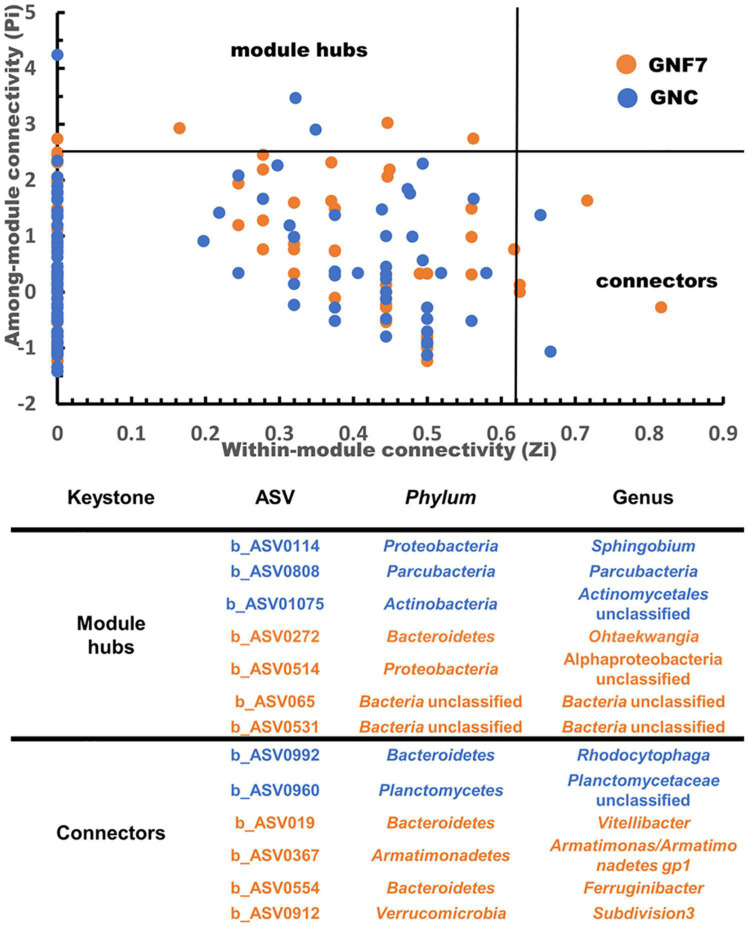
ZiPi plots highlighting the keystone amplicon sequence variants (ASV) of the banana (cv. Grand Nain) root microbial network of control (GNC) and *Pseudomanas simiae* PICF7-inoculated (GNF7) plants. The module hubs have a Zi value > 2.5 and Pi value ≤ 0.62, and the connectors have a Zi value ≤ 2.5 and Pi > 0.62. The details of each keystone ASV are shown in the table.

Microbial (bacteria and fungi) co-occurrence networks were constructed independently for the GNC and GNF7 plants, including all sampled time-points except 56 DAI. These networks exhibited a clear swift in the interaction pattern upon inoculation with the BCA (Figure 4). The results showed that the GNF7 network was less complex and more compartmentalized than the GNC network. This was confirmed by the topological network parameter values (Table 1)—for instance, the GNF7 network showed a smaller number of total links, a lower average degree (avgK) as well as a lower average clustering coefficient (avgCC) than GNC. However, the GNF7 co-occurrence network showed closer interactions or edges, represented as the average path distance (GD). This led us to infer that the GNF7 network components or ASV were less connected to their neighboring modules (Figure 4). Moreover, modularity was significantly higher in GNF7, although the number of modules was similar to that observed in the GNC samples. Overall, introduction of strain PICF7 altered the microbial interactions. Specifically, PICF7 interacted positively with three ASV—ASV0140 (*Gemmatimonas*), ASV0348 (*Candidatus* Saccharibacteria), and ASV0341 (*Ohtaekwangia*)—and negatively with two ASV—ASV017 (*Sphingopyxis*) and ASV055 (unclassified Chitinophagaceae). Furthermore, in the presence of PICF7, the network showed a larger percentage of positive interactions, increasing from 31% in GNC to 40% in GNF7 (Table 1). Additionally, the keystone taxa drastically changed upon PICF7 inoculation. GNF7 showed a greater number of module hubs and connectors (Figure 5). In this sense, the module hubs were classified at the genus level as *Ohtaekwangia*, two unclassified bacteria, and unclassified *Alphaproteobacteria*. In contrast, the GNC module hubs were represented by *Sphingobium*, *Parcubacteria*, and one unclassified Actinomycetales. Regarding connectors, GNF7 had four nodes classified as connectors, corresponding to the genera *Vitellibacter*, *Armatimonas*, *Ferruginibacter*, and one belonging to the phylum *Subdivision3*, while GNC had only two connectors, classified as *Rhodocytophaga* and an unclassified Planctomycetaceae. Remarkably, all module hubs and connectors were bacteria, and no fungal ASV was found as a keystone node (Figure 5).

**TABLE 1:** Main topological properties of co-occurrence networks of banana (cv. Grand Nain) root control (GNC) and *Pseudomanas simiae* PICF7-inoculated (GNF7) plants.

Community	No. of original ASV	Similarity thershold (St)	Total nodes	Total links	*R*^2^ of power-law	Average degree (avgK)	Average clustering coefficient (avgCC)	Average path distance (GD)	Modularity (no. of modules)	Percentage of positive edges
GNC	1,760	0.83	308	448	0.943	2.91	*0.079*	*6.277*	*0.715 (44)*	31%
GNF7	1,708	0.81	313	366	0.866	2.339	*0.071*	*6.004*	*0.778 (43)*	40%

*Significant coefficients (p-values < 0.02) between GNC and GNF7 plants are shown in italics. The numbers in brackets indicate modules that make up each network. ASV, amplicon sequence variants; GD, geodesic distance (or average path distance).*

### *Pseudomonas simiae* PICF7 Induced Transient Changes in the Expression of Banana Defense-Related Genes

**FIGURE 6:**
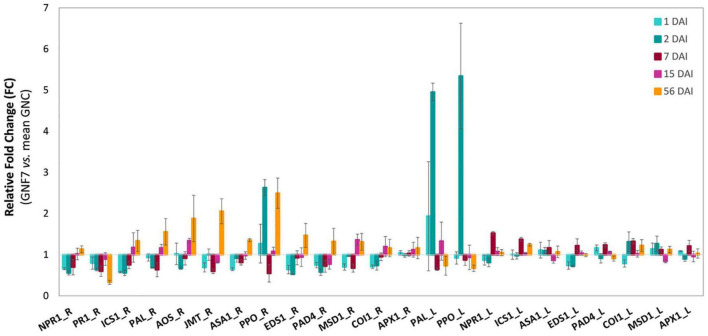
Relative expression levels (fold changes) of defense-related genes in banana plants upon inoculation with *Pseudomonas simiae* PICF7. Non-expresser of PR 1 (NPR1), pathogenesis-related 1 (PR1), isochorismate synthase 1 (ICS1), phenylalanine ammonia-lyase (PAL), allene oxide synthase (AOS), jasmonic acid carboxyl methyltransferase (JMT), anthranilate synthase alpha subunit 1 (ASA1), polyphenol oxidase (PPO), enhanced disease susceptibility 1 (EDS1), phytoalexin deficient 4 (PAD), manganese superoxide dismutase 1 (MSD1), coronatine insensitive 1 (COI1), and ascorbate peroxidase (APX1). FC < 1, downregulation; FC > 1, upregulation; R, roots; L, leaves. *Musa acuminata* genes *EF-1* and *L2* were used as internal controls to normalize the expression. Error bars, standard error of the mean (SEM); DAI, days after inoculation; GNC, non-inoculated plants; GNF7, *P. simiae* PICF7-inoculated plants. GNC/GNF7 = 4/4.

The expression pattern of 13 genes, each of them comprising between 1 and 9 *M. acuminata* paralogs, was analyzed in banana leaves and/or roots at 1, 2, 7, 15, and 56 DAI (Supplementary Table 5). In roots, seven genes (in)directly involved in SAR and/or ISR responses, namely, *NPR1*, *PR1*, *ICS1*, *PAL*, *AOS*, *JMT*, and *ASA1*, were significantly downregulated in at least one time-point during the first week upon inoculation with our BCA (Figure 6 and Supplementary Figures 5A–D). Among them, *PR1* also showed a significant downregulation at 56 DAI (Figure 6 and Supplementary Figure 5A). Repression at earlier time-points was also observed for genes *EDS1* and *PAD4*, involved in receptor-triggered immunity and salicylic acid (SA) accumulation, and for *MSD1*, related to mitochondrial ROS detoxification (Figure 6 and Supplementary Figures 5E,F). Conversely, induction of expression was observed at later time-points (15 and/or 56 DAI) for genes involved in SA biosynthesis (*PAL*), jasmonic acid (JA) biosynthesis (*AOS* and *JMT*), JA/ethylene-mediated defense (*ASA1*), or general plant defense (*PPO*) (Figure 6 and Supplementary Figures 5B–D). Interestingly, a significant upregulation was also observed for *PPO* at 2 DAI, indicating an early induction of this gene in roots (Supplementary Figure 5D). By contrast, *COI1* (defense-related) and *APX1* (ROS detoxification) did not show any significant change in root expression (Figure 6 and Supplementary Figures 5F,G). In leaves, the response was more limited to the first week upon inoculation with the BCA, with upregulation in four out of the 10 genes that were analyzed in this tissue, namely, *PAL* and *PPO* (induced at 2 DAI) and *NPR1* and *ICS1* (induced at 7 DAI; Figure 6 and Supplementary Figures 6A–E).

## Discussion

Plants live in intimate association with complex and diverse microbial communities, shaping an entity called holobiont (Bordenstein and Theis, 2015; Baedke et al., 2020). In this context, the impact of applying BCA should be evaluated taking into account not only their separated effects on the pathogen and/or the host but rather on the holobiont as a whole. Moreover, this multifaceted impact should be considered as an important criterion when assessing their suitability for commercial development. Therefore, modulation of the plant microbiome after the application of bio-inoculants is a relevant research question to be addressed. Recently, Berg et al. (2021) described six types of microbiome modulation: (i) transient microbiome changes, (ii) effect in microbial diversity (stabilization or increase), (iii) stabilization or increase of microbiome uniformity, (iv) restoration of microbial balance or reduction of a pathogen-triggered shift, (v) specific changes in a potential beneficial group of organisms, and (vi) depletion of potential pathogens. In order to include co-occurrence network shifts of plant microbiomes upon BCA introduction and according to our results, an additional modulation mechanism could be added to this list or, alternatively, one of the above-mentioned types could be redefined. In this sense, multi-omics approaches can contribute to better comprehend the microbial inoculant interaction with the holobiont (host, microbiome, and their interplay) (Jansson and Baker, 2016; Berg et al., 2021).

In this work, we studied the impact of *P. simiae* PICF7 on the banana holobiont under controlled conditions. We focused on possible changes on the microbial communities associated to the banana roots and on the potential priming and/or induction of certain genetic defense responses in the host (i.e., the macrobiont) upon PICF7 inoculation. One relevant result from this study was that the introduction of PICF7 did significantly alter neither the composition nor the structure of the banana root microbiota. This confers ecological advantage (i.e., minor impact on the resident microbiota) regarding future field applications of this BCA, which had been previously demonstrated to be effective against FWB (Gómez-Lama Cabanás et al., 2021). Only some differences at the ASV level are worth mentioning. Remarkably, although otherwise expected, ASV024 was identified as the introduced BCA only in the GNF7 samples. This ASV was observed at all time-points, although being more abundant at 1 and 2 DAI but almost vanished at 56 DAI. Low persistence of BCA inoculants and/or small impact on microbial communities after a relatively short period of time from their application have been previously reported (e.g., Yin et al., 2013; Chen et al., 2017; Wan et al., 2017; Jangir et al., 2019). Although PICF7 relative abundance declines at 1 week after inoculation, it seems that the biomass of the BCA is enough to exert its protective role as earlier demonstrated (Gómez-Lama Cabanás et al., 2021). ASV054, classified at the genus level as *Opitutus*, had a significantly higher abundance at 7 DAI in inoculated (GNF7) *vs.* non-inoculated (GNC) plants. Studies in other plant species such as *Lilium davidii* and *Cucumis sativus* showed an increase in *Opitutus* abundance in the microbiota of healthy plants as well as in plants with less symptoms of Fusarium wilt compared to plants showing a high disease severity (Shang et al., 2016; Jin et al., 2019). While these works focused on the interaction with a pathogen, it is tempting to speculate that the presence of PICF7 may favor the increase of potentially beneficial components of the banana root microbiota, thereby preparing the host to better counteract future challenges by the pathogen. A similar finding was observed for fungi. The relative abundance of ASV08, classified within the genus *Stachybotrys*, was significantly higher in GNF7 compared to the GNC plants at 56 DAI. Despite the fact that several species of this genus cause highly toxic mold to humans (Castlebury et al., 2004), *Stachytbotrys elegans* can be used as BCA since they act as mycoparasites of plant–pathogenic fungi like *Rhizoctonia solani* (Chamoun and Jabaji, 2011; Chamoun et al., 2013) and *Stachybotrys levispora* (Ribeiro et al., 2018). This information could be relevant, although these differences were only observed in one of the studied time-points (7 DAI for bacteria and 56 DAI for fungi), and further studies would be necessary to identify the precise species of *Opitutus* and *Stachybotrys* and the role that they may play in the banana root microbial community.

The most significant alterations upon PICF7 inoculation were found in the interactions of microbial communities through co-occurrence network analysis. In recent years, these kinds of analysis have proved to be more effective and sensitive than diversity indexes for studying the influence of external factors/perturbations in microbial communities (Barberán et al., 2012; Karimi et al., 2017; Kong et al., 2019; Benidire et al., 2020; Fernández-González et al., 2021). Among these perturbations, the infection by pathogens and the introduction of bio-inoculants (e.g., BCA) have shown to exert clear effects on microbial interaction patterns (Kong et al., 2019; Benidire et al., 2020; Fernández-González et al., 2020; Hu et al., 2020; Qu et al., 2020; Zheng et al., 2021). Our results confirmed the disturbance caused at this level when strain PICF7 was introduced. Indeed this BCA produced a drastic change in the topology of the network and hub taxa. More specifically, a decrease in network complexity when the bio-inoculant was present is evidenced by a decrease in avgCC, avgk, and the number of links (Table 1). Although PICF7 did not constitute a keystone in the banana root microbial network, it was identified as part of one of the main network modules in bacterized plants. Thus, this bacterium seems to play an important role in shaping the community network. Some studies have reported an increase in the connections of networks when a pathogen is present (Fernández-González et al., 2020; Hu et al., 2020). It is tempting to speculate that the reduction of connections observed upon PICF7 inoculation (Table 1 and Figure 4), together with the increase in modularity, could somehow undermine a subsequent interaction with the pathogen, minimizing its negative effects. However, testing this hypothesis would require a further investigation. In fact, the increase in modularity has been earlier proposed as a way to stabilize networks, helping to prevent disease development by means of compartmentalization of the pathogen (Delmas et al., 2019). In addition, a decrease in GD was observed for GNF7 *vs*. GNC (Table 1). A lower GD has been related to a higher global efficiency (Strang et al., 2018). Thus, an ecosystem with a smaller GD can have more efficient interactions among its nodes, thereby providing higher stability to the ecosystem in the presence of perturbations (Tao et al., 2018; Qu et al., 2020).

As mentioned earlier, the identification of keystone taxa (i.e., nodes with highest interactions between modules or within its own modules) can be instrumental for recognizing new BCA. Additionally, it was previously reported that these keystone taxa change in response to external conditions (Tao et al., 2018). This is in agreement with the results presented here, in which module hubs and connectors changed in number and taxonomy depending on the tested conditions (Figure 5). Once again, this fact highlights strain PICF7 as sharply influencing the community interaction patterns of the network of inoculated plants. While in the GNF7 network four module hubs and four connectors were identified, only three module hubs and two connectors were observed in the GNC network (Figure 5). Among the module hubs identified in the GNF7 plants, a representative of the genus *Ohtaekwangia* (ASV0272) is worth mentioning. Members of this genus have been described to produce compounds with antibiotic and antifungal properties, a fact that could benefit the community when fighting against pathogens (Okanya et al., 2011). In relation to this, ASV024 (i.e., PICF7) was interacting with five other nodes (Figure 4), and, among them, a positive correlation was found with a member of the genus *Ohtaekwangia.* This finding, along with the identification of an *Ohtaekwangia* member as a keystone taxon, points out to the fact that this genus could be a promising source of new bio-inoculants or as a member of synthetic communities designed to control FWB. However, the ASV belonging to *Ohtaekwangia* genus (ASV0341) on which PICF7 had a positive effect was different to the ASV previously mentioned as module hub in the GNF7 network. Further studies would thus be needed in order to identify and isolate members of this genus as potential new BCA or to be used in combination with PICF7, taking advantage of potential synergistic effects. Bacteria belonging to the genus *Gemmatimonas* could likewise be investigated based on their positive interactions with strain PICF7. *Candidatus* Saccaribacteria, in contrast, while showing positive interactions, cannot be used in such a way due to its unculturable nature, at least at the moment.

The influence of *P. simiae* PICF7 on the microbial community co-occurrence network was evaluated by combining the samples of all time-points except for 56 DAI, when the PICF7 relative abundance was significantly lower compared to the previous time-points. Remarkably, the PICF7 relative abundance in the GNF7 plants reached its highest value at 2 DAI. However, the relative abundance of the BCA quickly decreased at 7 DAI and nearly faded out at 56 DAI. Therefore, the influence of PICF7 on the banana root co-occurrence network could be assumed to be crucial only at earlier moments after the introduction of the BCA.

Another interesting aspect of this study was the ability of PICF7 to mount defense responses in banana plants, particularly at the root level. Therefore, the effective biocontrol activity displayed by PICF7 against FWB (*Foc* STR4) described in our previous work (Gómez-Lama Cabanás et al., 2021) could be explained, at least partly, by a combination of early modifications on the indigenous microbial network and changes in the expression of defense-related genes. The main objective of our time-course expression analysis was to investigate the induction of both local and systemic responses which could potentially lead to a primed state of the plants. Priming is an adaptive, low-cost strategy that improves the defensive capacity of plants when they are later challenged by a triggering stimulus (i.e., pathogen attack) (Conrath et al., 2015). Upon perception of this challenge, defense is deployed in a faster, stronger, and/or more sustained manner, and this landmark can even be transmitted to subsequent generations constituting a type of plant immunological memory (Luna et al., 2012). Holistic approaches have demonstrated that priming stimuli trigger direct changes in the plant which are crucial for the enhanced defensive behavior (Balmer et al., 2015), with no or minimal fitness costs associated in terms of growth or seed/fruit production. It is reported that beneficial members of the root microbiota are able to induce systemic resistance and trigger defense priming (Pieterse et al., 2014). However, during the early stages of colonization, they try to suppress plant immunity, a strategy that allows them to establish a more prolonged and intimate interaction with their host (Zamioudis and Pieterse, 2012; Pascale et al., 2020). Eventually, these mutualists would be recognized as non-hostile, thus activating host immunity and priming the plant for future infections (Zamioudis and Pieterse, 2012). In line with this, our time-course expression analysis showed an initial suppression of a number of SAR- and ISR-related genes upon inoculation with *P. simiae* PICF7 (Figure 6 and Supplementary Figures 5, 6), suggesting that this strain tried to evade the banana immune responses during the first phase of the interaction with the plant. Moreover, a transcriptomic study involving *P. simiae* WCS417, a nearly isogenic strain of PICF7 (Montes-Osuna et al., 2021), proved a wide downregulation of immune responses in *A. thaliana* roots during early colonization by the former BCA (Stringlis et al., 2017). These results suggested that the initial suppression of host defense would be a mechanism generally used by this bacterium when colonizing different plant species. Remarkably, Morán-Diez et al. (2012) also showed downregulation of genes related to JA- and SA-mediated defense early after inoculating *A. thaliana* plants with the beneficial fungus *Trichoderma harzianum* T34. This response included a strong repression of *EDS1* and *PR1*, two of our selected genes, followed by their later upregulation and priming of host defense. Interestingly, the time-point when PICF7 relative abundance reached its highest values (i.e., 2 DAI; Figure 3) corresponded to a significant repression of *AOS*, *EDS1*, *ICS1*, *NPR1*, *PAD4*, and *PR1* in roots, while *PPO* showed an induced expression. *PPO* was likewise upregulated in leaves at this particular time-point, indicating the activation of a transient systemic response in banana during the first week after inoculation with *P. simiae* PICF7.

In summary, the impact of strain PICF7 on the banana holobiont should be considered as an important criterion (and added value) when assessing their suitability for field trials and commercial development. Here we found that this BCA did not decisively alter the indigenous banana root microbiota since no permanent effect on the community composition was observed. Despite that the PICF7 relative abundance reached the highest values soon after being inoculated, followed by a rapid decline, it is noteworthy that this BCA was detected as a component of one of the main modules in the microbial co-occurrence network. Furthermore, its presence produced a dramatic shift in the interactions among components of the banana root microbiota, an outcome evidenced by significant differences in the topology of the network. This fact, together with the induction of key defense-related genes and its beneficial effect on decreasing FWB severity caused by STR4 (Gómez-Lama Cabanás et al., 2021), makes PICF7 a promising and “low-environmental-impact” candidate for developing a successful BCA formulation. Besides this, microbial ecological network studies like the one reported here can provide relevant information for the development of new BCA inoculants or to highlight new candidates for generating synthetic communities. Nonetheless, due to their importance, microbiome changes upon BCA inoculation definitively require a better understanding of the underlying communication and interaction mechanisms within the holobiont, both in the presence and absence of different *Foc* races. Moreover, further experimental evidence under field conditions will be needed to decipher these complex questions and validate the results previously obtained under controlled conditions.

## Data Availability Statement

The datasets presented in this article can be found in online repositories. The names of the repository/repositories and accession number can be found at: https://www.ncbi.nlm.nih.gov/, PRJNA747136.

## Author Contributions

JM-B conceived the study. CG-LC and JM-B designed the experiments. CG-LC and AV-C carried out the sampling. CG-LC performed all the experiments. AV-C contributed to the execution of all the experiments. YZ-F performed the analysis of gene expression. NW, AF-G, and MF-L performed the bioinformatics and statistical analyses of NGS data. CG-LC, JM-B, YZ-F, and NW wrote the manuscript. All authors have read and agreed to the published version of the manuscript.

## Conflict of Interest

The authors declare that the research was conducted in the absence of any commercial or financial relationships that could be construed as a potential conflict of interest.

## Publisher’s Note

All claims expressed in this article are solely those of the authors and do not necessarily represent those of their affiliated organizations, or those of the publisher, the editors and the reviewers. Any product that may be evaluated in this article, or claim that may be made by its manufacturer, is not guaranteed or endorsed by the publisher.

## Footnotes

1
https://benjjneb.github.io/dada2/tutorial.html

2
https://benjjneb.github.io/dada2/ITS_workflow.html

3
https://plutof.ut.ee/#/doi/10.15156/BIO/786343

4
https://www.ncbi.nlm.nih.gov

5
http://ieg4.rccc.ou.edu/mena/main.cgi

6
https://www.arabidopsis.org/

7
http://primer3.ut.ee/

8
https://www.qbaseplus.com
