# Supplementary material for: Impacts of the Biocontrol Strain Pseudomonas simiae PICF7 on the Banana Holobiont: Alteration of Root Microbial Co-occurrence Networks and Effect on Host Defense Responses
Source: Front Microbiol. 2022 Feb 15;13:809126. doi: 10.3389/fmicb.2022.809126 (PMC8885582; doi:10.3389/fmicb.2022.809126)
Supplement: Supplementary file 1 [file Data_Sheet_1.docx]

Supplementary Material

**
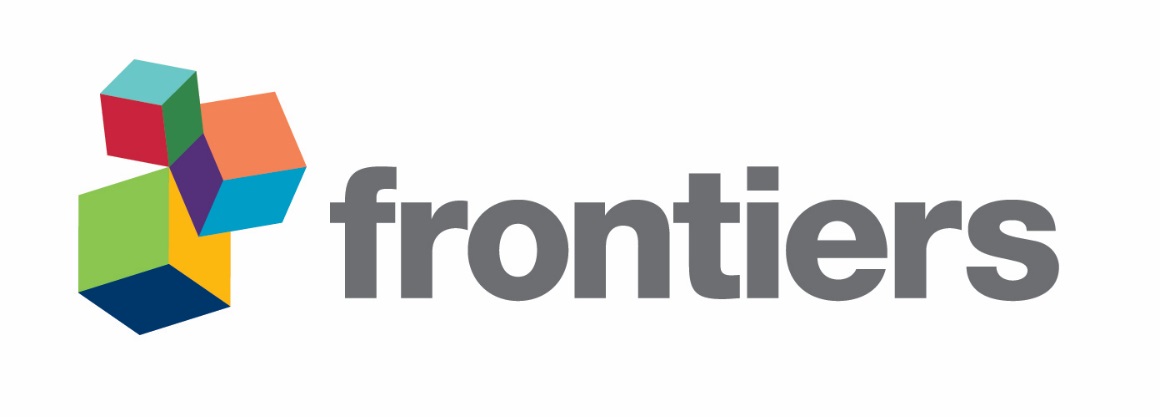
**

**Supplementary Figure 1.** Principal coordinates analyses of the banana root microbial communities. PCoA of bacterial (A) and fungal (B) communities by treatment on Bray-Curtis dissimilarities. GNC: control plant, GNF7: plant inoculated with *Pseudomonas simiae* PICF7, DAI: days after inoculation.


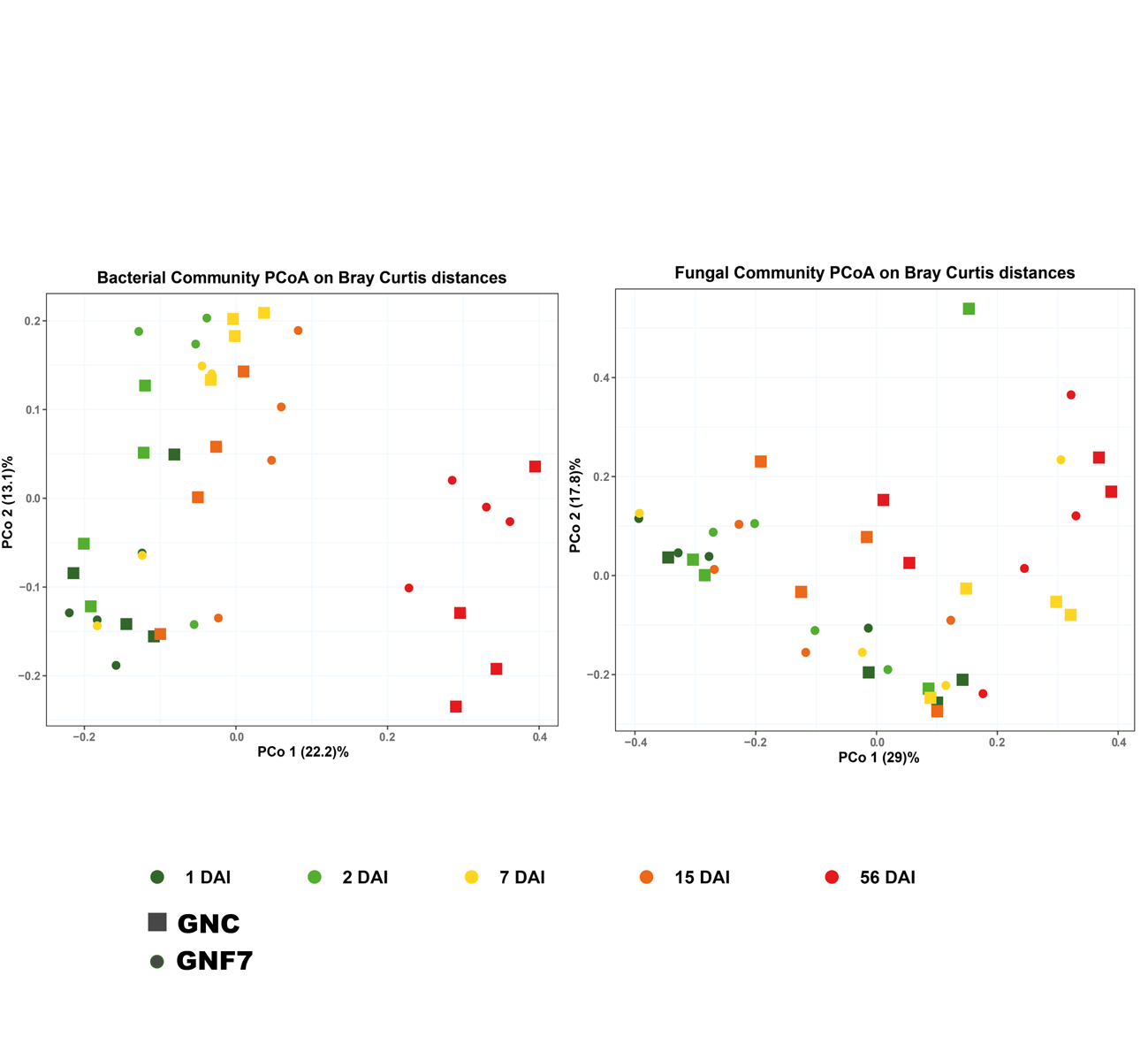


**Supplementary Figure 2.** The rarefaction curve. The abscissa is the number of sampling sequences and the ordinate is the number of Amplicon Sequence Variants (ASV) in the sampling sequences. Different colors indicate different groups. Black color indicates control and inoculated plants (GNC and GNF7) and orange color Grand Nain *in vitro* plants (GNI). The ASV 0 to 100 range has been magnified to show differences.


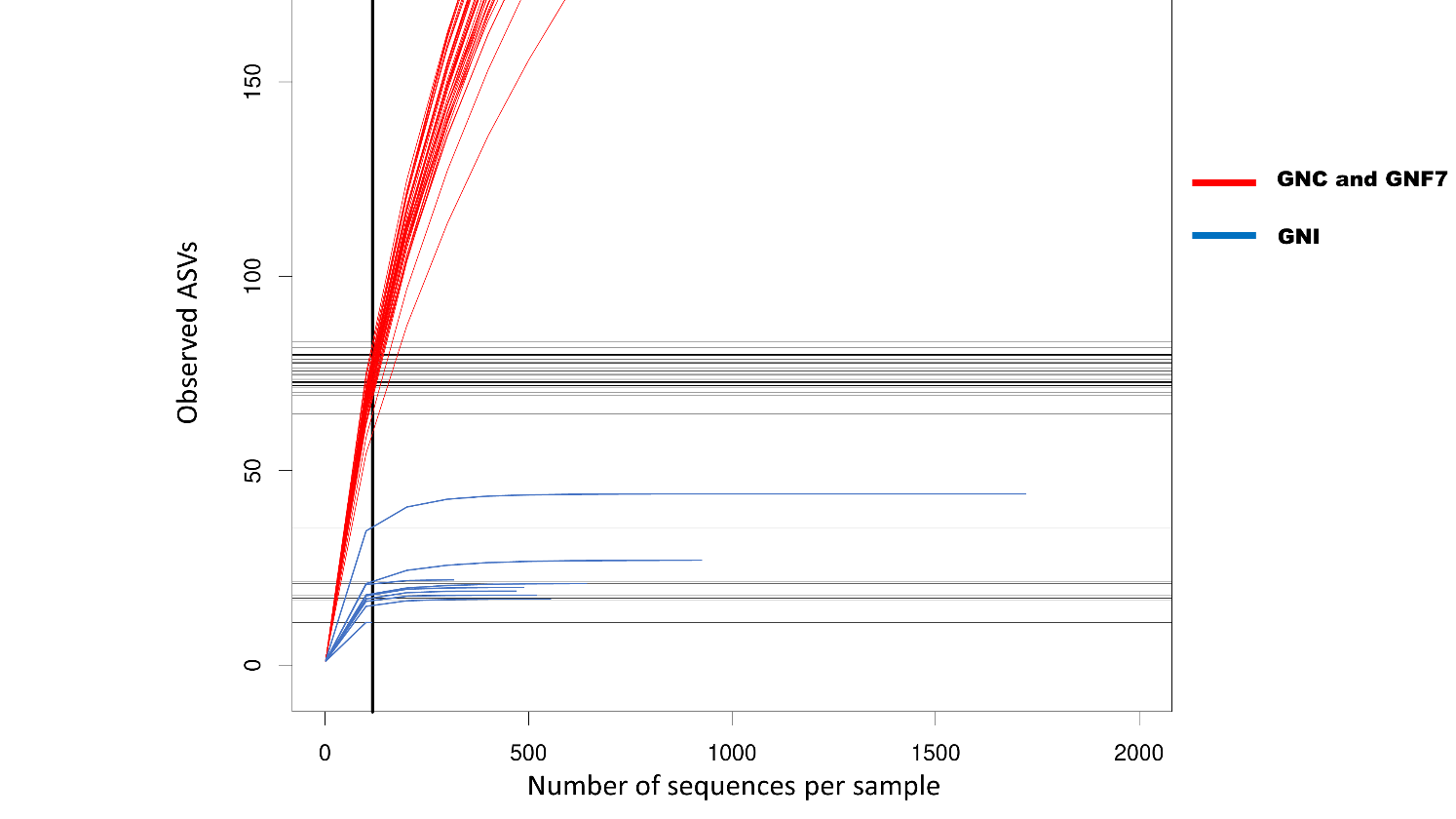


**Supplementary Figure 3.** Box plot of α-diversity indices: InvSimpson, observed Amplicon Sequence Variants (ASV) and Shannon for the fungal community comparing Grand Nain *in vitro* (GNI), control (GNC) and *Pseudomanas simiae* PICF7-inoculated (GNF7) plants.


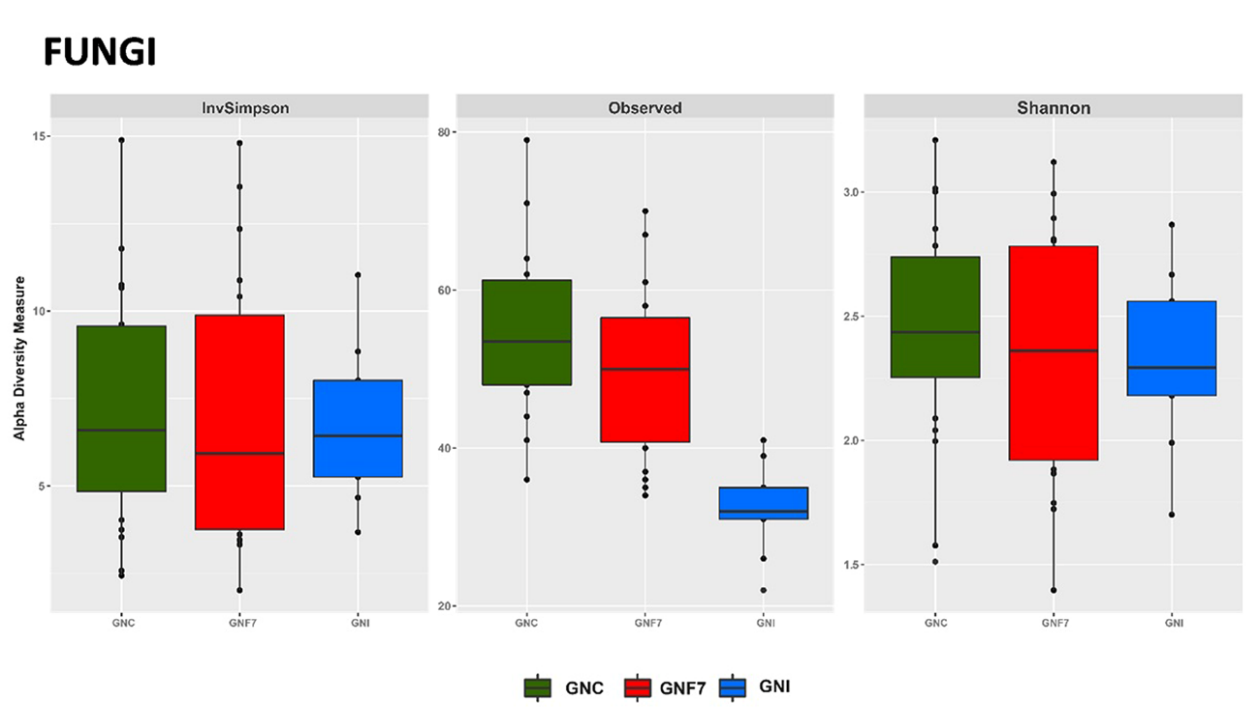


**Supplementary Figure 4.** Principal coordinates analyses of the banana root microbial communities. PCoA of bacterial by treatment on Weighted Unifrac distances (A) and fungal communities by treatment on Bray-Curtis dissimilarities (B). GNI: Grand Nain *in vitro*, GNC: control plant, GNF7: plant inoculated with *Pseudomonas simiae* PICF7.


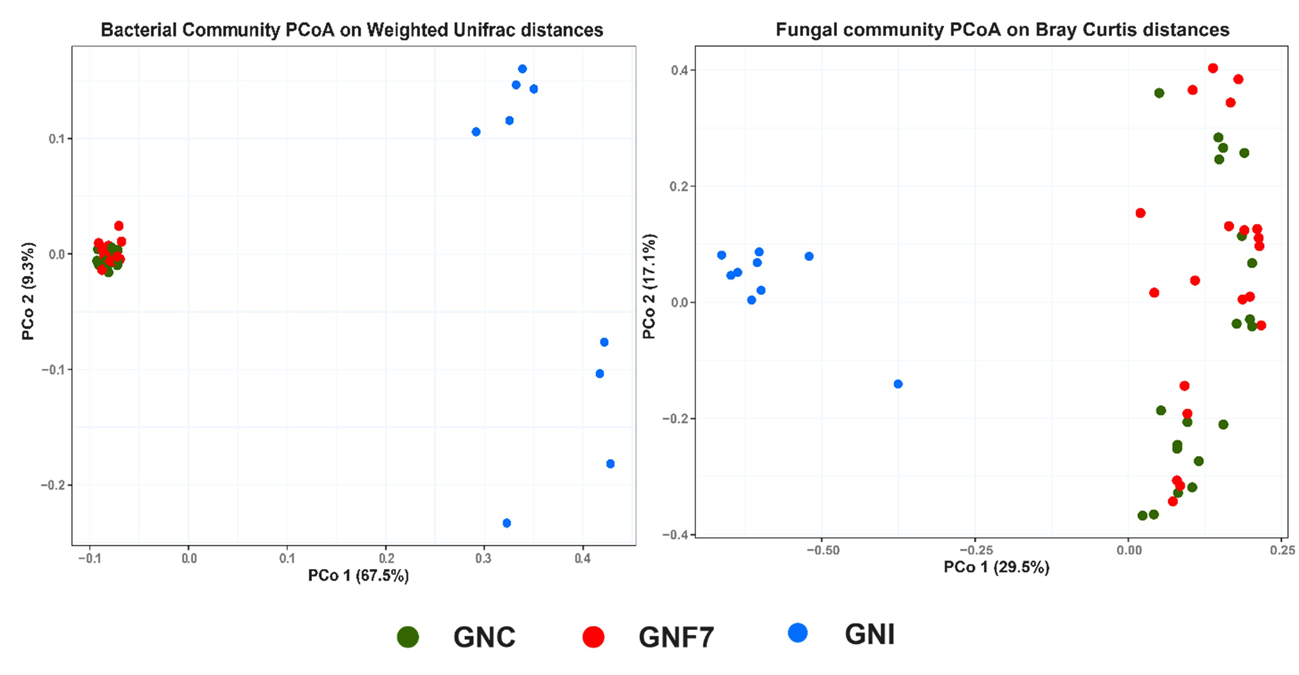


**Supplementary Figure 5.** Relative expression levels of defence-related genes in roots at five time points upon inoculation with *Pseudomonas simiae* PICF7. (A) *Non-expresser of PR genes 1* (*NPR1*) and *Pathogenesis-related 1* (*PR1*); (B) *Isochorismate synthase 1* (*ICS1*) and *Phenylalanine ammonia-lyase* (*PAL*); (C) *Allene oxide synthase* (*AOS*) and *Jasmonic acid carboxyl methyltransferase* (*JMT*); (D) *Anthranilate synthase alpha subunit 1* (*ASA1*) and *Polyphenol oxidase* (*PPO*); (E) *Enhanced disease susceptibility 1* (*EDS1*) and *Phytoalexin deficient 4* (*PAD4*)*;* (F) *Manganese superoxide dismutase 1* (*MSD1)* and *Coronatine insensitive 1* (*COI1*); (G) *Ascorbate peroxidase* (*APX1*). CNRQ: Calibrated Normalized Relative Quantity. *Musa acuminata* genes *EF-1* and *L2* were used as internal controls to normalize the expression data. Letters in black and bold type indicate significant differences based on comparison over treatments and time points (LSD; α = 0.05). Letters in grey indicate non-significant differences. Error bars: standard error of the mean (SEM). DAI: days after inoculation. GNC: non-inoculated plants; GNF7: *Pseudomonas simiae* PICF7-inoculated plants. N control/inoculated=4/4.


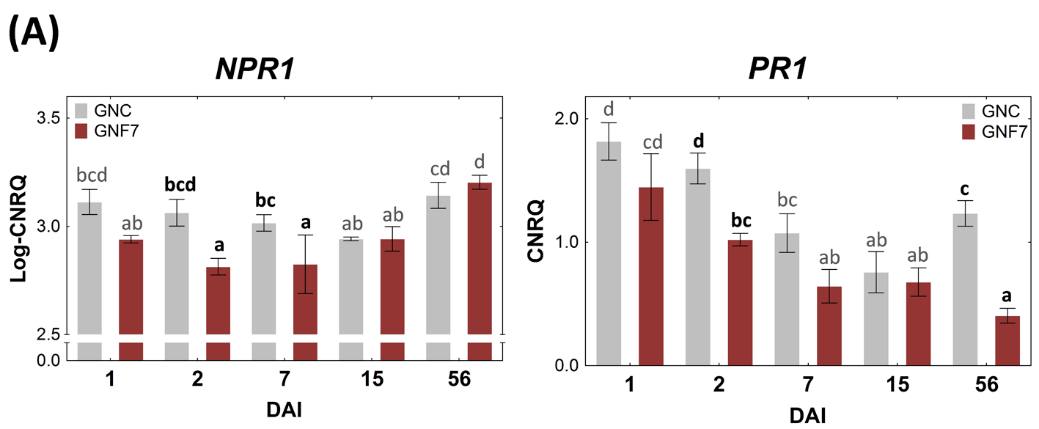


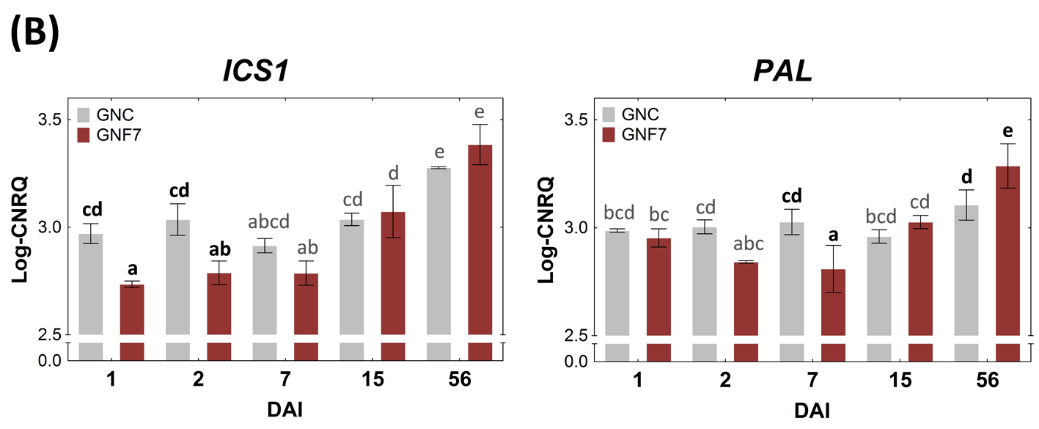


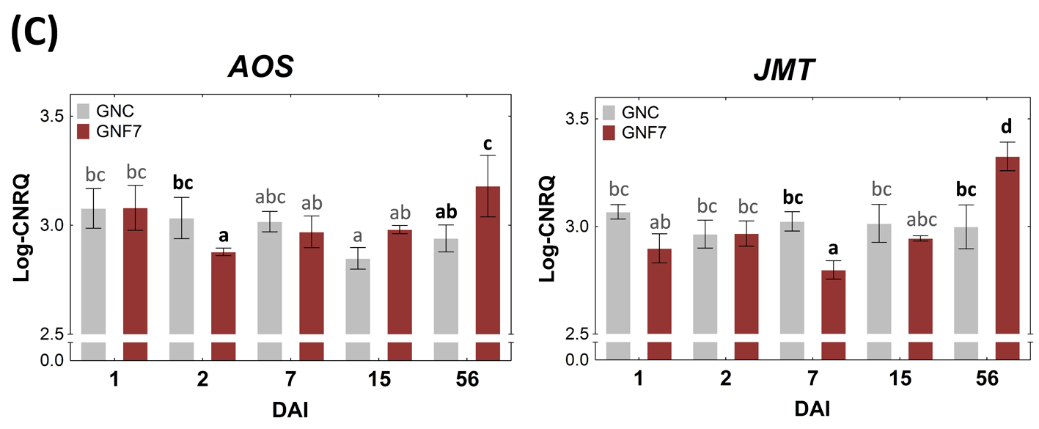


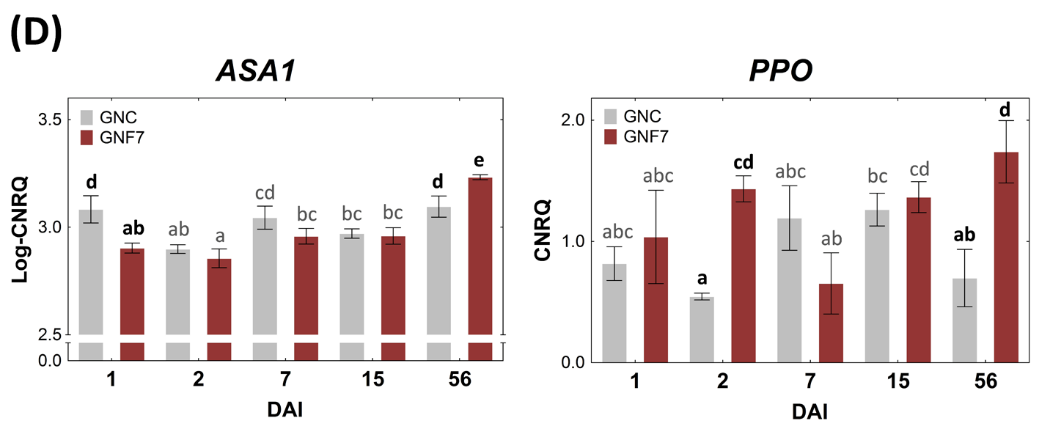


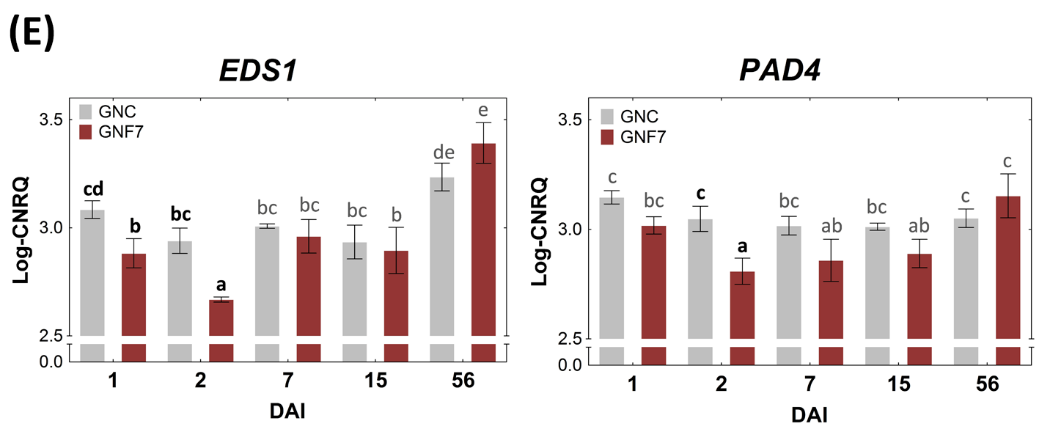


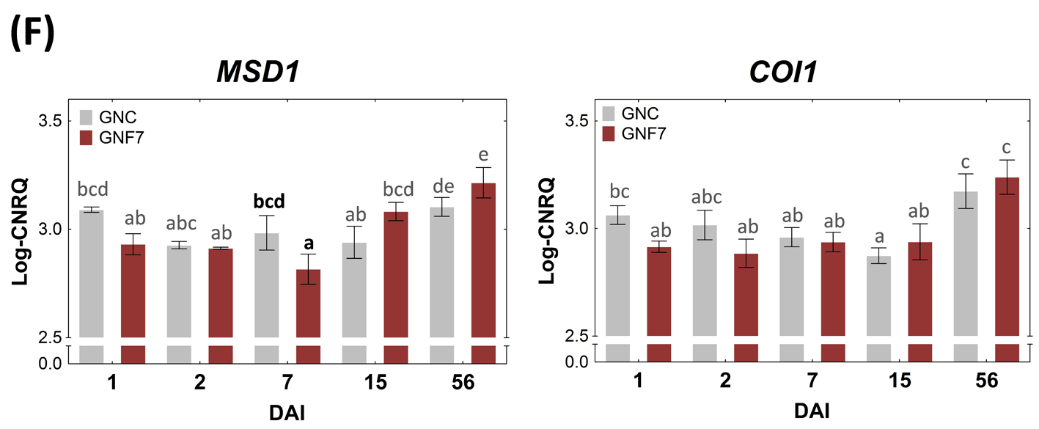


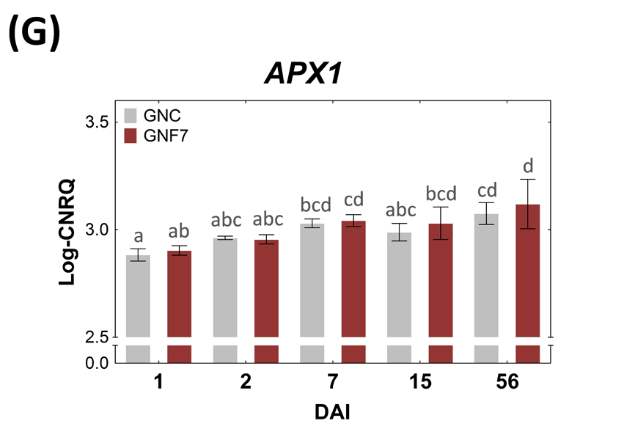


**Supplementary Figure 6.** Relative expression levels of defence-related genes in leaves at five time points upon inoculation with *Pseudomonas simiae* PICF7. (A) *Non-expresser of PR genes 1* (*NPR1*) and *Isochorismate synthase 1* (*ICS1*), (B) *Enhanced disease susceptibility 1* (*EDS1*) and *Phytoalexin deficient* (*PAD4*), (C) *Coronatine insensitive 1* (*COI1*) and *Anthranilate synthase alpha subunit 1* (*ASA1*), (D) *Phenylalanine ammonia-lyase* (*PAL*) and *Polyphenol oxidase* (*PPO*), (E) *Ascorbate peroxidase* (*APX1*) and *Manganese superoxide dismutase 1* (*MSD1*). CNRQ: Calibrated Normalized Relative Quantity. *Musa acuminata* genes *EF-1* and *L2* were used as internal controls to normalize the expression data. Letters in black and bold type indicate significant differences based on comparison over treatments and time points (LSD; α = 0.05). Letters in grey indicate non-significant differences. Error bars: standard error of the mean (SEM). DAI: days after inoculation. GNC: non-inoculated plants; GNF7: *Pseudomonas simiae* PICF7-inoculated plants. N control/inoculated=4/4.


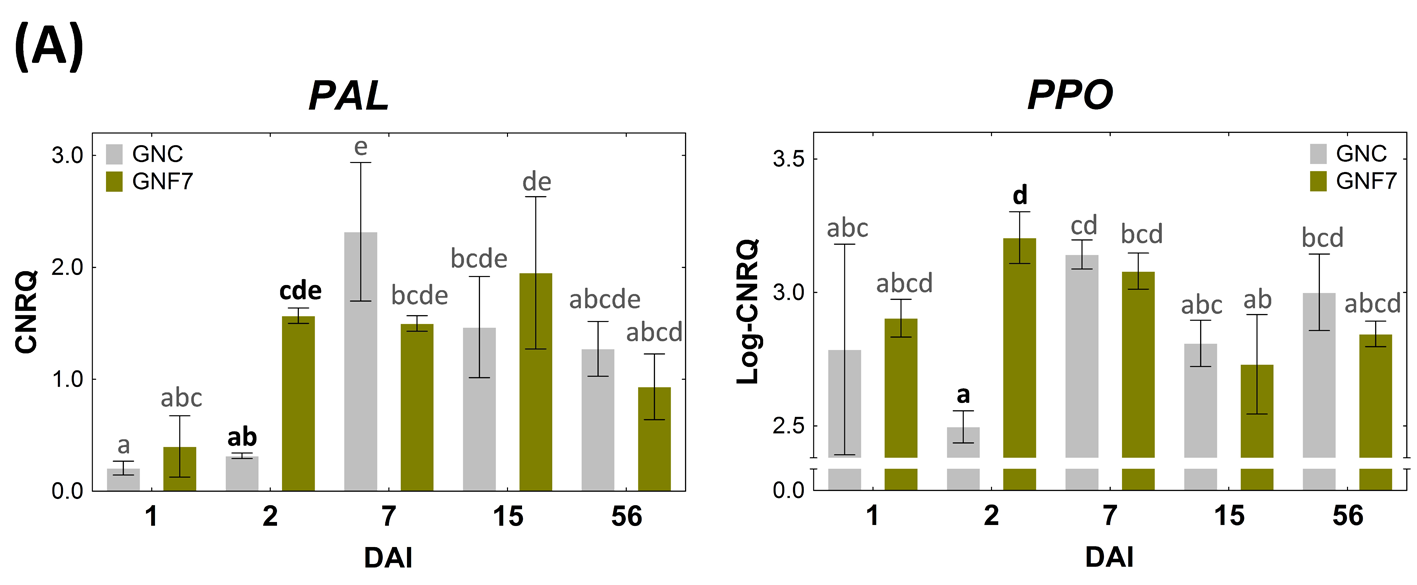


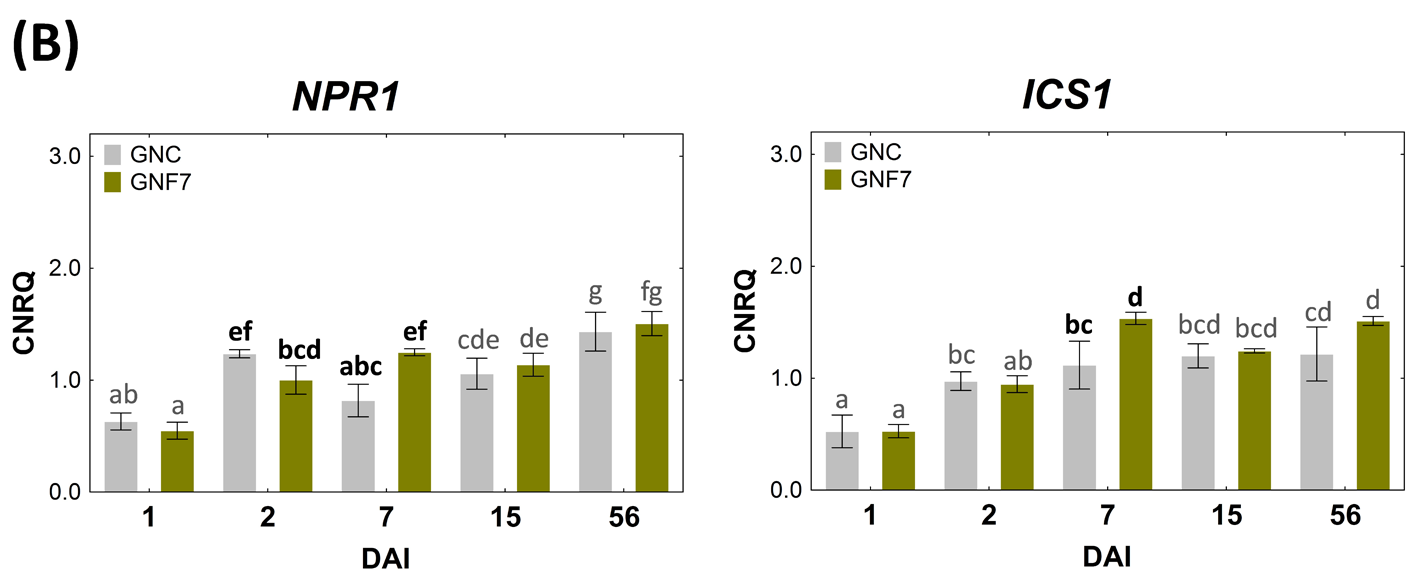


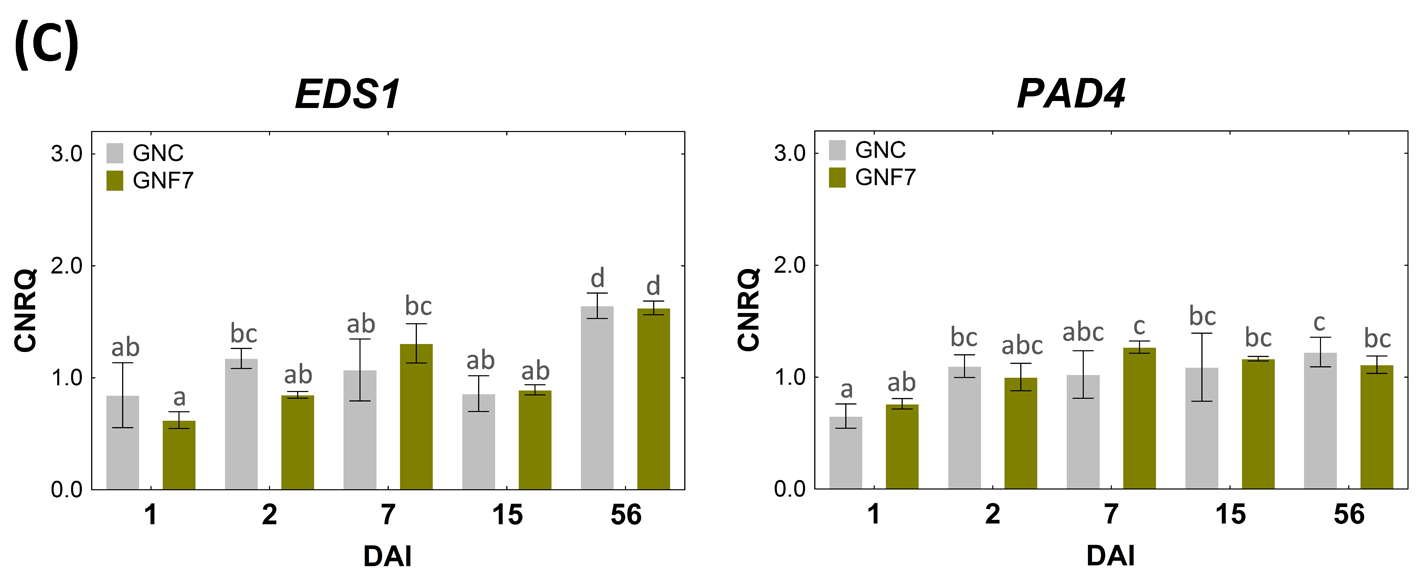


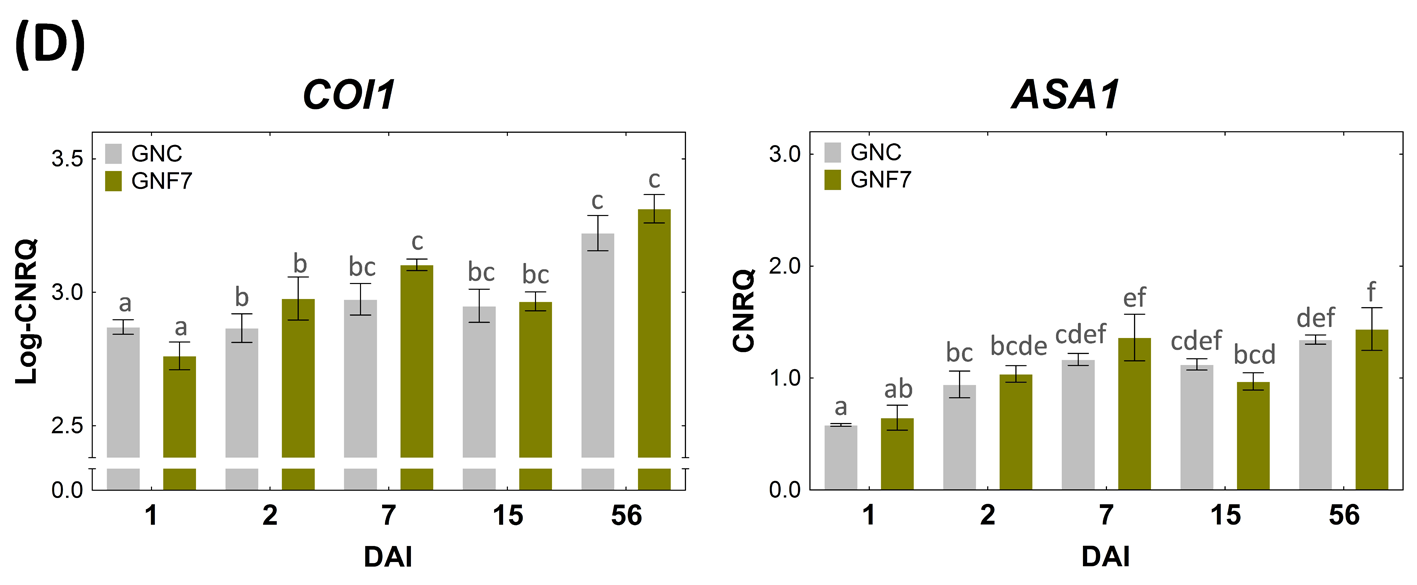


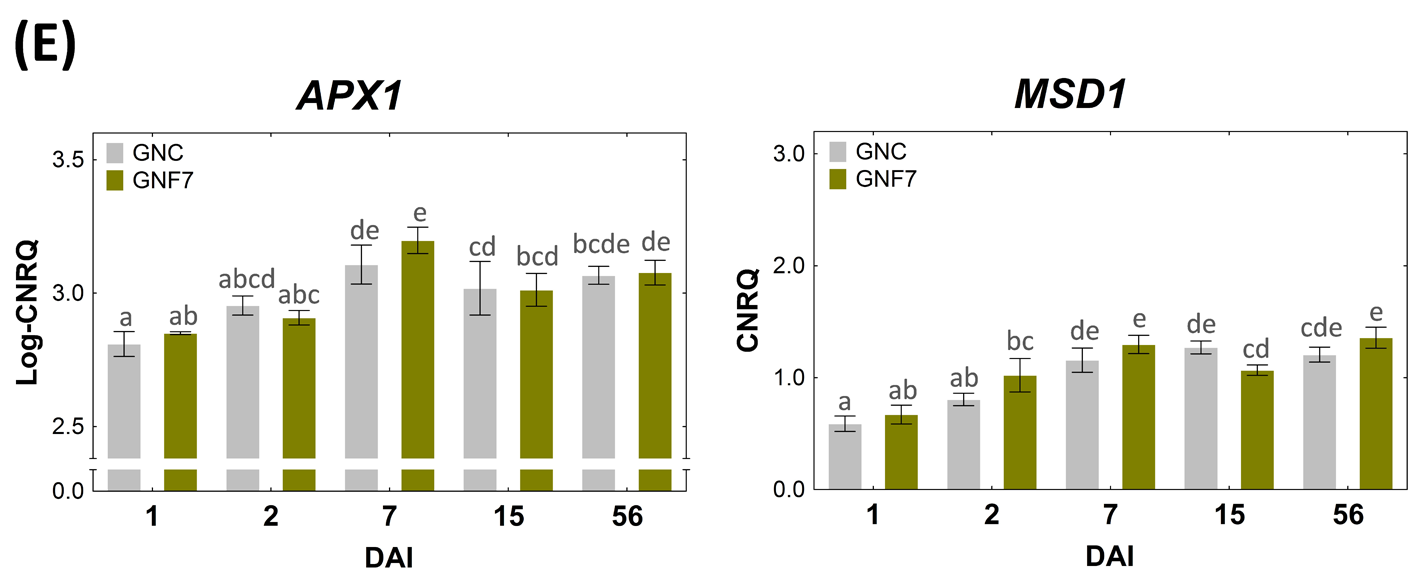


**Supplementary Tables**

**Supplementary Table S1.** Taxonomical profile of Amplicon Sequence Variants (ASV) present in at least 4 of the 10 Grand Nain *in vitro* samples analysed.

| **ASV** | **Kingdom** | **Phylum** | **Genus** | **Rel. abundance (%)** |
| --- | --- | --- | --- | --- |
| ASV0245 | *Bacteria* | *Proteobacteria* | *Sphingomonas* | 8.452 |
| ASV0788 | *Bacteria* | *Bacteria_unclassified* | *Bacteria_unclassified* | 1.398 |
| ASV01013 | *Bacteria* | *Proteobacteria* | *Halomonas* | 1.145 |
| ASV01094 | *Bacteria* | *Bacteria_unclassified* | *Bacteria_unclassified* | 0.879 |
| ASV056 | *Bacteria* | *Actinobacteria* | *Mycobacterium* | 0.769 |
| ASV01284 | *Bacteria* | *Bacteroidetes* | *Chitinophagaceae_unclassified* | 0.736 |
| ASV01216 | *Bacteria* | *Bacteria_unclassified* | *Bacteria_unclassified* | 0.601 |
| ASV01360 | *Bacteria* | *Firmicutes* | *Erysipelothrix* | 0.563 |
| ASV01493 | *Bacteria* | *Bacteria_unclassified* | *Bacteria_unclassified* | 0.531 |
| ASV01410 | *Bacteria* | *Proteobacteria* | *Acinetobacter* | 0.467 |
| ASV01311 | *Bacteria* | *Firmicutes* | *Bacillus* | 0.465 |
| ASV01359 | *Bacteria* | *Proteobacteria* | *Pseudomonas* | 0.448 |
| ASV01580 | *Bacteria* | *Actinobacteria* | *Propionibacterium* | 0.428 |
| ASV01527 | *Bacteria* | *Proteobacteria* | *Halomonas* | 0.421 |
| ASV01805 | *Bacteria* | *Proteobacteria* | *Pseudomonas* | 0.363 |
| ASV01784 | *Bacteria* | *Bacteria_unclassified* | *Bacteria_unclassified* | 0.333 |
| ASV01600 | *Bacteria* | *Bacteria_unclassified* | *Bacteria_unclassified* | 0.331 |
| ASV02671 | *Bacteria* | *Bacteroidetes* | *Flavobacteriaceae_unclassified* | 0.228 |
| ASV0407 | *Bacteria* | *Proteobacteria* | *Escherichia/Shigella* | 0.210 |
| ASV02485 | *Bacteria* | *Bacteria_unclassified* | *Bacteria_unclassified* | 0.158 |
| ASV016 | *Fungi* | *Ascomycota* | *Pseudogymnoascus* | 22.082 |
| ASV018 | *Fungi* | *Ascomycota* | *Geomyces* | 18.586 |
| ASV027 | *Fungi* | *unclassified* | *unclassified* | 16.310 |
| ASV044 | *Fungi* | *Ascomycota* | *Mycosphaerella* | 6.083 |
| ASV060 | *Fungi* | *Ascomycota* | *Cladosporium* | 3.085 |
| ASV09 | *Fungi* | *Ascomycota* | *Phialemonium* | 1.029 |
| ASV0123 | *Fungi* | *Basidiomycota* | *unclassified* | 0.757 |
| ASV0147 | *Fungi* | *Basidiomycota* | *unclassified* | 0.407 |
| ASV0195 | *Fungi* | *Ascomycota* | *unclassified* | 0.321 |
| ASV0250 | *Fungi* | *Basidiomycota* | *unclassified* | 0.191 |
| ASV0317 | *Fungi* | *unclassified* | *unclassified* | 0.123 |
| ASV0283 | *Fungi* | *unclassified* | *unclassified* | 0.081 |
| ASV0392 | *Fungi* | *unclassified* | *unclassified* | 0.075 |

**Supplementary Table S2.** *p*-values of bacterial and fungal richness (Observed Amplicon Sequence Variants; ASV), Shannon and InvSimpsom α-diversity indices for the different comparisons analysed. Significant *p*-values and its correspondent significant comparisons are shown in bold type and italics. GNI: Grand Nain *in vitro* plants, GNC: control plants, GNF7: *Pseudomonas simiae*-inoculated plants.

|  | **Observed ASV** | | **Shannon** | | **InvSimpson** | |
| --- | --- | --- | --- | --- | --- | --- |
| **Comparison** | **Bacteria** | **Fungi** | **Bacteria** | **Fungi** | **Bacteria** | **Fungi** |
| GNC vs. GNF7 time to time | ***0.014*** | ***0.018*** | 0.745 | 0.219 | 0.568 | 0.166 |
| GNC along time | 0.072 | ***0.024*** | 0.970 | ***0.020*** | 0.612 | ***0.015*** |
| GNF7 along time | 0.172 | 0.140  ***0.140*** | 0.805 | 0.803  0.803 | 0.540 | 0.623  ***0.623*** |
| GNI vs. GNC vs. GNF7 | *-* | ***<0.001*** | - | ***0.68*** | - | ***0.94*** |

**Supplementary Table S3.** *p*-values of PERMANOVA and BETADISPER analysis of quantitative β-diversity index for the different comparisons analysed. GNI: Grand Nain *in vitro* plants, GNC: control plants, GNF7*: Pseudomonas simiae*-inoculated plants. Significant p-values and its correspondent significant comparisons are shown in bold type and italics.

|  | **PERMANOVA *p-value*** | | **BETADISPER *p-value*** | |
| --- | --- | --- | --- | --- |
| **Comparison** | **Bacteria** | **Fungi** | **Bacteria** | **Fungi** |
| GNC vs. GNF7 time to time | ***0.001*** | ***0.002*** | 0.758 | 0.31 |
| GNC along time | ***0.001*** | ***0.016*** | 0.171 | 0.778 |
| GNF7 along time | ***0.001*** | ***0.017*** | 0.988 | 0.145 |
| GNI vs. GNC and GNF7 | ***0.001*** | ***0.001*** | ***0.001*** | 0.153 |

**Supplementary Table S4.** *Pseudomonas simiae* strain PICF7 (GenBank: CP005975.1) *16S rRNA* gene sequence comparison with Amplicon Sequence Variants (ASV) belonging to *Pseudomonas* species found in Grand Nain Control and Grand Nain PICF7 samples.

**Supplementary Table S5.** List of defence related genes studied by real-time PCR experiments in root and aerial tissues of banana plants (cv. Grand Nain) grown under control conditions upon *Pseudomonas simiae* PICF7 inoculation. For all transcripts, Relative expression analysis was repeated at least two times in independent (4 replicates each one) real-time qPCR experiments. Gene names, abbreviation, *Arabidopsis thaliana* orthologs, *Musa acuminata* paralogs, primers sequences, amplicon size, annealing temperature, PCR efficiencies, correlation coefficients (R2) and linear equations are indicated. Fw: forward, Rv: reverse.
